# Comparison of *Mycoplasma pneumoniae* Genome Sequences from Strains Isolated from Symptomatic and Asymptomatic Patients

**DOI:** 10.3389/fmicb.2016.01701

**Published:** 2016-10-27

**Authors:** Emiel B. M. Spuesens, Rutger W. W. Brouwer, Kristin H. J. M. Mol, Theo Hoogenboezem, Christel E. M. Kockx, Ruud Jansen, Wilfred F. J. Van IJcken, Annemarie M. C. Van Rossum, Cornelis Vink

**Affiliations:** ^1^Division of Infectious Diseases and Immunology, Department of Pediatrics, Erasmus MC-Sophia Children's HospitalRotterdam, Netherlands; ^2^Center for Biomics, Erasmus MCRotterdam, Netherlands; ^3^Laboratory of Pediatrics, Department of Pediatrics, Erasmus MC-Sophia Children's HospitalRotterdam, Netherlands; ^4^Regional Laboratory of Public Health Kennemerland, Department of Molecular BiologyHaarlem, Netherlands; ^5^Department of Life Sciences, Erasmus University CollegeRotterdam, Netherlands

**Keywords:** *Mycoplasma pneumoniae*, whole genome sequencing, infection, colonization, respiratory tract infections

## Abstract

*Mycoplasma pneumoniae* is a common cause of respiratory tract infections (RTIs) in children. We recently demonstrated that this bacterium can be carried asymptomatically in the respiratory tract of children. To identify potential genetic differences between *M. pneumoniae* strains that are carried asymptomatically and those that cause symptomatic infections, we performed whole-genome sequence analysis of 20 *M. pneumoniae* strains. The analyzed strains included 3 reference strains, 3 strains isolated from asymptomatic children, 13 strains isolated from clinically well-defined patients suffering from an upper (*n* = 4) or lower (*n* = 9) RTI, and one strain isolated from a follow-up patient who recently recovered from an RTI. The obtained sequences were each compared to the sequences of the reference strains. To find differences between strains isolated from asymptomatic and symptomatic individuals, a variant comparison was performed between the different groups of strains. Irrespective of the group (asymptomatic vs. symptomatic) from which the strains originated, subtype 1 and subtype 2 strains formed separate clusters. We could not identify a specific genotype associated with *M. pneumoniae* virulence. However, we found marked genetic differences between clinical isolates and the reference strains, which indicated that the latter strains may not be regarded as appropriate representatives of circulating *M. pneumoniae* strains.

## Introduction

*Mycoplasma pneumoniae* is a human bacterial pathogen that has been estimated to cause pneumonia in up to 40% of children hospitalized because of community-acquired pneumonia (Waites and Talkington, 2004). In addition, *M. pneumoniae* is associated with extra-pulmonary manifestations of which the most common are central nervous system complications (e.g., Guillain-Barré Syndrome and encephalitis), haemolytic anemia, non-specific myalgias or arthralgias, and renal complications, such as glomerulonephritis. *M. pneumoniae* infections are commonly treated with antibiotics that target the bacterial DNA metabolism or protein synthesis. However, evidence for effectiveness of these antibiotics against *M. pneumoniae* infections is scarce and based on comparative studies (Mulholland et al., 2012).

A recent study has shown that *M. pneumoniae* can also be carried asymptomatically: the bacterium was detected in the upper respiratory tract of 21% of children that did not show any signs of a respiratory tract infection (RTI) (Spuesens et al., 2013). Asymptomatic carriage of potential pathogens is a well-known, common phenomenon in the general population. For instance, potential pathogens like *Streptococcus pneumoniae, Staphylococcus aureu*s and rhinovirus can cause RTIs, but can also be carried asymptomatically. Clearly, it is important to identify the determining factors, both from pathogen and host, involved either in control of an asymptomatic carriage status or in triggering symptomatic infection. In the case of *S. pneumoniae*, specific bacterial gene clusters were found to be associated with invasive disease (Wulff-Burchfield et al., 2010). Several *M. pneumoniae* virulence factors have been identified in previous studies (Waites and Talkington, 2004). The first step to invasive disease is the attachment of *M. pneumoniae* to its human host using an elaborate attachment organelle, which consists of several attachment and accessory proteins. Without an intact attachment organelle, *M. pneumoniae* is unable to cause disease. Several other virulence mechanisms include direct cytotoxicity (e.g., by production of H_2_O_2_) and activation of the inflammatory cascade leading to cytokine-mediated tissue injury (Waites and Talkington, 2004). In 2005, the Community-Acquired Respiratory Distress Syndrome (CARDS) toxin was discovered as another potential virulence factor (Kannan et al., 2011). It is unknown whether all *M. pneumoniae* strains express these factors or whether there are genes in *M. pneumoniae* associated with carriage and/or pathogenicity.

Until a few years ago, only a single genome sequence was available for *M. pneumonia* (Himmelreich et al., 1996). This sequence was derived from laboratory strain M129. Since 2013, several other genomic sequences have been published (Lluch-Senar et al., 2015; Simmons et al., 2013; Xiao et al., 2015). Most of these sequences were obtained in two independent studies. One of these studies reported the comparative genome analysis of 15 *M. pneumoniae* strains isolated form respiratory tract samples and cerebrospinal fluid samples collected from patients between 1940 and 2009 in the USA, China, and England (Xiao et al., 2015). The other study reported the sequences of 23 clinical *M. pneumoniae* strains isolated between 1964 and 2011 in six different countries (Lluch-Senar et al., 2015). In both studies, the genomes of *M. pneumoniae* strains seem very stable over time and at different locations in the world. The sequences of these studies show a low number of non-synonymous single-nucleotide polymorphisms, but a high rate of variation among repetitive elements (Lluch-Senar et al., 2015; Xiao et al., 2015).

Despite the availability of a significant set of *M. pneumoniae* genome sequences, it is currently difficult to determine the association between *M. pneumoniae* genotype and virulence. This is mainly due to the fact that all known *M. pneumoniae* sequences were exclusively obtained from strains isolated from patients with RTI symptoms. Clearly, this precludes determination of the putative genetic differences between strains causing symptomatic infections and strains carried by asymptomatic individuals. The recent isolation of a set of *M. pneumoniae* strains from both asymptomatic children and children suffering from RTI (Spuesens et al., 2013), however, allows a direct comparison of the genetic make-up of strains associated with bacterial carriage and those involved in symptomatic infection. We therefore set out to determine the genome sequence of 3 reference strains (M129, FH, and R003), 3 strains from asymptomatic individuals, and 13 strains from clinically well-defined patients suffering from either an upper or lower RTI. The analysis and comparison of the obtained sequences did not reveal a specific genotype associated with *M. pneumoniae* virulence. However, we did find striking differences between the genomes of clinical isolates and those of the *M. pneumoniae* reference strains.

## Methods and materials

### Patient samples

Most of the patient samples were collected as part of a clinical study designed to investigate the existence of *M. pneumoniae* asymptomatic carriage in children (Spuesens et al., 2013). This study was carried out in Rotterdam, the Netherlands between 2008 and 2012. Patient information was collected and documented prospectively as part of the study. All *M. pneumoniae* culture-positive samples from the different groups (asymptomatic, symptomatic, and follow-up) were selected for analysis as part of this study. The other samples from symptomatic patients were collected by the Regional Laboratory of Public Health Kennemerland, Haarlem, The Netherlands. These samples were taken from patients with suspected *M. pneumoniae* infection as part of the medical work-up ordered by the treating physician. All samples with a positive culture for *M. pneumoniae* were selected and used in this study. The Medical Ethics Review Board of the Erasmus MC approved the study on asymptomatic carriage (NL20418.078.08) and written informed consent was obtained in this study from all parents and children above the age of 12 years. The Medical Ethics Review Board of the Erasmus MC approved the use of the samples collected during routine medical work-up in the Regional Laboratory of Public Health Kennemerland (MEC 2013-344).

### Culture and DNA isolation

The culturing of *M. pneumoniae* was performed in the laboratory of pediatrics of the Erasmus MC, as previously described (Sluijter et al., 2008). In short, 100 μl original sample and 10-fold dilutions were used for culturing. Culturing was performed in *Mycoplasma* medium containing 1.4% Difco™ PPLO broth (Becton Dickinson), 0.15% Difco™ TC Yeastolate, UF (Becton Dickinson), 1.4% glucose, 20% horse serum, 1,000 U/ml Penicillin G, 500 U/ml Polymyxine B, and 0.02 mg/ml phenol red. The pH of the medium was adjusted to 7.8–8.0 using a solution of 2 N NaOH, followed by filter-sterilization. Cells were harvested upon color change of the medium (from red/orange to yellow). The cells were added to Mycoplasma medium agar plates and single colonies were harvested. These were grown in 3 ml of medium at 37°C/5% CO_2_ in 25 cm^2^ tissue culture flasks (Greiner). Cells were harvested upon color change of the medium, and DNA was isolated from the cells as described previously (Spuesens et al., 2009).

### Grouping of the strains

The strains were divided into 4 groups as indicated in Table 1. Group 1 includes reference strains *M. pneumoniae* M129 (subtype 1, ATCC 29342), *M. pneumoniae* FH (subtype 2, ATCC 15531) and *M. pneumoniae* R003 (subtype 2a). The other groups consisted of strains isolated from asymptomatic children (Group 2), patients with an upper RTI (Group 3) and patients with a lower RTI (Group 4).

**Table 1:** **Sample ID, patient information, strain information and ***de novo*** assembly information**.

**Group**	**Strain/sample ID**	**Diagnose group**	**Sequence ID**	**Size (bp)^2^**	**Subtype^c^**	**Largest contig (bp)**	**N50 contig (bp)**	**M129 genome covered (%)^d^**
1	M129	Reference	01	802,479	1	135,172	59,602	99.89
	FH	Reference	02	803,911	2	98,770	50,959	99.29
	R003	Reference	03	800,612	2	123,538	54,253	99.21
2	B174	Asymptomatic	11	805,944	1	135,177	53,512	99.83
	B247	Asymptomatic	12	801,799	1	96,961	66,934	99.63
	B406	Asymptomatic	14	804,626	2	98,962	51,557	99.06
3	A016	Upper RTI	04	800,489	2	123,550	54,265	99.18
	A058	Upper RTI	06	800,399	1	135,171	54,703	99.73
	C024	Upper RTI	15	809,160	1	135,170	59,355	99.85
	H030	Upper RTI	19	806,820	1	135,258	59,621	99.81
4	A035	Lower RTI	05	805,945	2	123,540	54,255	99.04
	A103	Lower RTI	07	821,841	1	92,532	45,886	99.73
	HAP111	Lower RTI	10	808,319	2	135,217	54,427	99.23
	HAP157	Lower RTI	09	800,436	1	92,731	51,728	99.73
	H010	Lower RTI	18	805,135	1	191,508	66,444	99.67
	H016	Lower RTI	20	805,824	1	135,161	59,300	99.77
	H026	Lower RTI	13	790,390	2	98,587	48,754	98.80
	C036-1^a^	Lower RTI	16	807,258	1	135,171	59,440	99.77
	C036-2^a^	Lower RTI	17	804,586	1	191,498	66,641	99.73
	HAP157FUP	Follow-up	08	802,814	1	191,496	66,615	99.75

a *Morphologically different M. pneumoniae colonies originating from the same patient sample. Both a large (C036-1) and a small colony (C036-2) were sequenced*.

b *Total assembly (bp). Contigs over 500 bp in length are considered in the assembly size*.

c *Subtype 1 and 2 as described previously by Spuesens et al. (2013)*.

d *All contigs, including contigs below 500 bp in length, were aligned to the M129 reference genome with MUMmer*.

### Sequencing

The sequencing of all strains including re-sequencing of the reference strains was performed at the Center for Biomics of the Erasmus MC using an Illumina HiSeq2000 sequencer. DNA libraries were prepared according to the Illumina TruSeq DNA protocol. The libraries were sequenced using the TruSeq V2 protocol with paired-end 100-bp reads. Between 0.9 and 2.5 gigabases of DNA sequence was generated for each of the isolates and a 1224- to 3300-fold genome coverage was obtained. The generated short-read datasets were submitted to the NCBI Sequence Read Archive (SRA) under accession number SRP081446.

### *De novo* assembly

To check the quality of the raw sequence data we used FastQC. This analysis showed that the majority of the sequenced data had a PHRED quality score exceeding Q30, which is considered as good quality (Supplementary File 1). Reads were purged from the Illumina sequence adapter and renamed according to the standards expected by Abyss (Software Abyss 1.3.6). Bases with a PHRED quality score below 30 (base call accuracy ≥ 99.9%) were not used in the assembly. After the initial processing, Abyss was run for each of the samples individually. For each sample, the optimal k-mer was determined by varying k between 25 and 100 in steps of 5 (Supplementary Figure 1). The assembly with the largest N50 contig was considered optimal.

The assembled sequences were separately reported by Abyss as unitigs, contigs, and scaffolds. The unitig files hold the assemblies that were generated without taking the pair information into account. The contigs are the assemblies generated with the pair information taken into account. Scaffolds consist of merged contigs based on read pairs, and differ from the contigs in that they may contain unresolved repeats and spacers. For the downstream analysis, the contigs from the assemblies with the largest N50 contigs were used. These contigs are presented in Supplementary File 3.

Larger rearrangements, insertions and deletions were determined by comparing the assembled contigs with the *M. pneumoniae* M129 reference genome (NCBI accession number NC_000912.1). This alignment was performed with MUMmer (version 3.23) (Kurtz et al., 2004). These alignments are presented in Supplementary File 2. Analysis of the alignments was performed in R (version 3.2.2).

### SNV and InDel comparisons

In addition to the *de novo* assembly, the reads were aligned to the *M. pneumoniae* M129 reference genome (NCBI accession number NC_000912.1) using BWA (Li and Durbin, 2009) (version 0.5.9) to detect smaller variants. Single-nucleotide variants (SNVs) and short insertions and deletions (InDels) were determined relative to the reference strain with SAMtools mpileup (Li et al., 2009) (version 0.1.16) (Supplementary Table 1 and Supplementary File 4). For each sample, the frequencies of the SNVs and InDels relative to the total number of reads at that position were determined as well.

The strains were clustered based on their SNV/InDel profiles. SNV/InDels that were not present in at least 20% of the reads at a position in a single strain were removed from the analysis. These filtered SNV/InDels were considered low-abundant and may be caused by intrastrain variations or technical errors. The strains were then clustered based on their SNV/InDels profiles using hierarchical clustering. The distances between the SNV/InDels profiles were calculated with the Euclidean distance measure. These analyses were performed with standard facilities present in R (version 3.2.2) (Kurtz et al., 2004; Wickham, 2009; R Development Core Team, 2011; de Vries and Ripley, 2015).

## Results

### Selection of strains and isolates of *M. pneumoniae*

We have determined the complete genome sequences of a total of 20 strains or isolates of *M. pneumoniae*. The isolates were obtained either from asymptomatic children (*n* = 3), from patients with an upper RTI (URTI; *n* = 4), or from patients with a lower RTI (LRTI; *n* = 9). The names (ID) and origins of the isolates are listed in Table 1. Two of the isolates from the LRTI group were obtained at the same time from a single patient (C036-1 and C036-2). These two isolates were selected because they differed in colony morphology on agar plates. Two other samples from the LRTI group were also collected from a single patient, one at the moment of RTI (HAP157) and one collected 4 weeks later, after recovery of the clinical symptoms (HAP157FUP). In addition to the clinical isolates, three reference laboratory strains were included in this study, i.e., subtype 1 strain M129, and subtype 2 strains FH and R003.

### DNA sequencing and *de novo* genome sequence assembly

The genomic sequences of the 20 *M. pneumoniae* strains were determined using a paired-end 100-bp sequencing protocol on the HiSeq2000 platform (Illumina). For each of the strains, genome assemblies were generated that ranged from 790 kb (H026) to 821 kb (A103) in length (Table 1). These cumulative contig lengths are similar to the published genome length of reference strain M129 (816 kb) (Himmelreich et al., 1996). For strain M129, which was also included in our study, the total amount of sequence contained in contigs over 500 bp was found to be ~802 kb, which is 14 kb shorter than the previously published genome size of this strain (Table 1). Thus, ~2% of the M129 genome could not be reliably retrieved with the procedures used in this study. When contigs under 500 bp were considered as well, 99.9% of the M129 genome was covered (Table 1; Supplementary Table 1). The gaps are likely caused by repetitive sequences (RepMP sequences) (Spuesens et al., 2009) in the genome of *M. pneumoniae*, which are known to pose problems in short read sequencing. Gaps similar to those in the M129 genome were also found in the other strains (Table 1). Strikingly, the type 1 strains covered ~0.5% of the M129 genome more than the type 2 strains did. This difference is centered around a single locus at position 558,624–561,515, which is present in type 1 strains but is absent from type 2 strains (Supplementary File 2).

We identified one sequence stretch within the assembled contigs that was exclusively present in type 2 strains. The length of this stretch is 5.3 kb (in strains R003, A016, and A035) or 5.7 kb (in strains FH, B406, HAP111, and H026). The 5.3-kb fragment completely overlaps with the 5.7-kb fragment and corresponds to the sequence at position 704,213–709,505 of the FH genome. The 5.7-kb fragment corresponds to position 703,813–709,505 of this genome.

### Sequence differences between strains isolated from asymptomatic and symptomatic individuals

**Figure 1:**
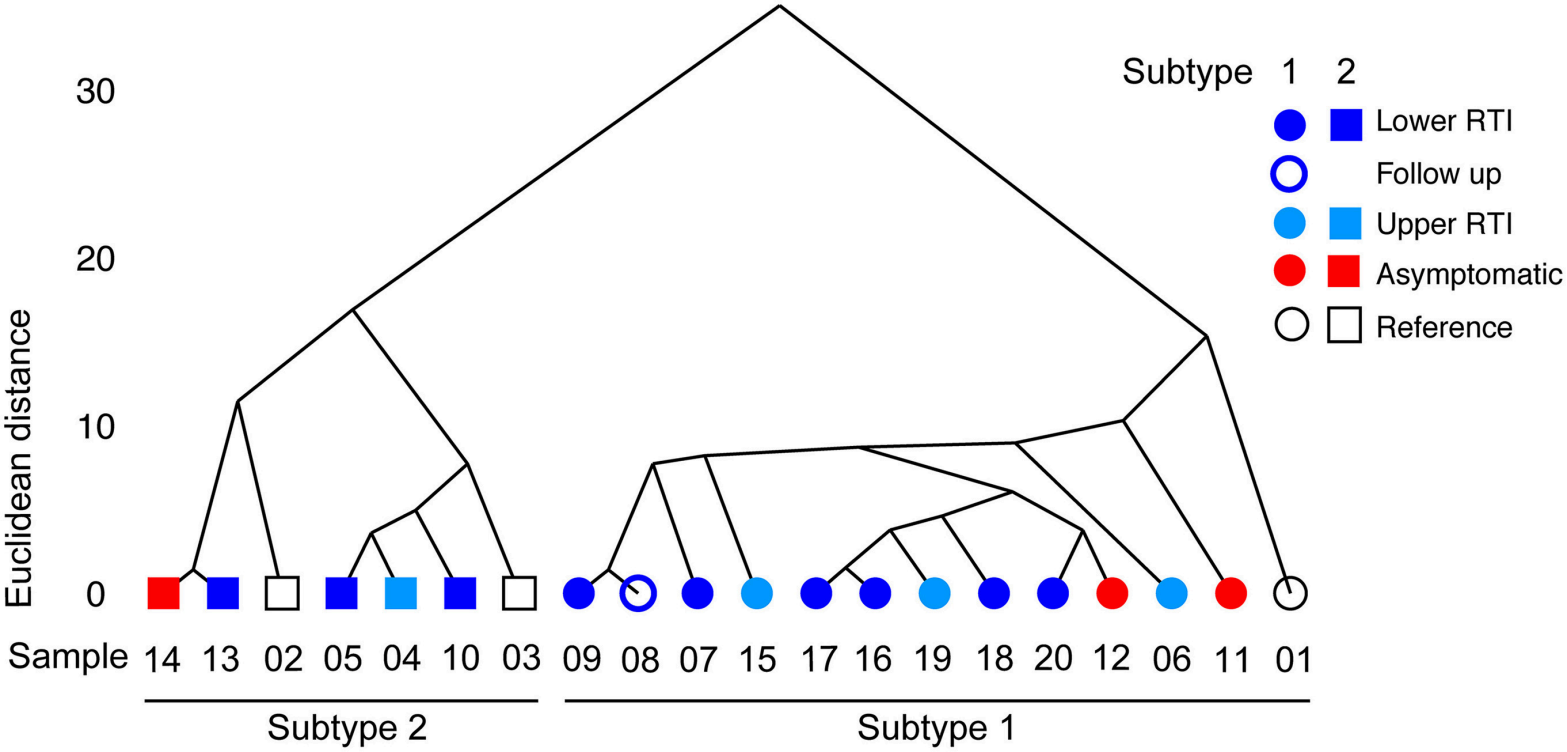
**Hierarchical clustering based on Euclidean distance**. The Euclidean distance between variant frequencies (on the y-axis) is plotted against the different strains sequenced in this study. The strains are indicated by their sequence ID listed in Table 1. Only the variants with a frequency above 0.2 were used in this analysis (see the text for details). Hierarchical clustering was applied based on Euclidean distance and the average linkage method (hclust).

To compare the genomic sequences of the different *M. pneumoniae* strains, their SNV/InDels profiles were subjected to hierarchical clustering analysis (Figure 1). This analysis clearly distinguished two major families of strains, which correspond to the known major subtypes of *M. pneumoniae*, i.e., subtypes 1 and 2. However, genetically similar strains did not cluster together with regard to the clinical groups of patients they were isolated from (Groups 1–4 in Table 1). For example, strain B406, which was isolated form an asymptomatic child (Group 2), shows a high degree of similarity with strain H026, which was isolated from an adult with an LRTI (Group 4). The hierarchical clustering based on SNV/InDels therefore does not indicate that the overall genome similarity is greater among the strains within each group than between strains from different groups.

To investigate whether the strains within each of the four groups share specific single nucleotide polymorphisms, small insertions or deletions in their genomes, a subtractive comparative approach was performed in which the following criteria were applied: (1) all variants with a low coverage (less than 21 times covered sequence) are filtered in each sample, (2) SNV/InDels present in the asymptomatic group are filtered from those in the other groups except the reference group (since SNV/InDels in the asymptomatic group are unlikely to cause a “phenotype”), (3) all SNV/InDels present in the reference group are filtered from those in the other groups except the asymptomatic group, and (4) only SNV/InDels are analyzed that are present in all samples in one group. Using this procedure, we were unable to identify variants that were shared between all samples from the same group. Although a number of variants were shared between some samples from the same group (Table 2), we did not find specific sequence differences between the strains from the group of symptomatic patients (Groups 3 and 4) and those from the other groups (Groups 1 and 2). Variant frequencies for the different comparisons are shown in Tables 2–**4**.

Table 2**SNV/InDels frequencies of the isolates from symptomatic groups**.

**Table:** 

**Upper RTI group**					
**SNV/InDels**	**Sequence ID**				**Locus**
**(NC_000912.1)**	**05**	**07**	**09**	**10**	
111954-111954:C-A	0.28	0.00	0.24	0.20	MPN089
528806-528806:G-GT	0.38	0.42	0.33	0.05	NA
536132-536132:A-ACC	0.67	0.00	0.00	0.71	MPN442
622601-622601:G-GTT	0.38	0.40	0.10	0.33	NA
706408-706408:A-ACC	0.21	0.01	0.00	0.22	NA

**Table:** 

**Lower RTI group**										
**SNV/InDels**	**Sequence ID**									**Locus**
**(NC_000912.1)**	**05**	**07**	**09**	**10**	**13**	**16**	**17**	**18**	**20**	
41006-41006:G-A	0.00	0.00	0.00	0.00	0.00	0.99	1.00	1.00	0.00	MPN034
140960-140960:C-A	0.00	0.00	0.00	0.00	0.00	1.00	0.99	1.00	0.00	MPN108
171528-171528:A-AG	0.00	0.00	0.00	0.00	0.00	0.67	0.68	0.66	0.00	MPN132
171529-171529:C-CCCAAG	0.00	0.00	0.00	0.00	0.00	0.66	0.67	0.65	0.00	MPN132
405692-405692:A-T	0.00	0.00	0.00	0.00	0.00	0.99	0.99	1.00	0.00	MPN341
428005-428005:A-G	0.00	0.00	0.00	0.00	0.00	1.00	0.99	1.00	0.00	MPN358
536132-536132:A-ACC	0.68	0.00	0.00	0.01	0.00	0.73	0.70	0.71	0.67	MPN442
622601-622601:G-GTT	0.18	0.06	0.40	0.39	0.01	0.04	0.06	0.30	0.33	NA
733651-733651:G-A	0.00	0.00	0.00	0.00	0.00	0.99	0.99	1.00	0.00	MPN612

*NA, Not applicable*.

### Variation in follow-up samples of a single patient

Two of the isolates that were sequenced were acquired at two different time-points from the same patient. The first isolate, HAP157, was obtained when this patient suffered from an LRTI. The other isolate, HAP157FUP, was taken 4 weeks later, after resolution of the infection following a course of antibiotics (azithromycin). Eight relevant differences were found between strains HAP157 and HAP157FUP (Table 3). One of these differences was localized to the P1 gene (MPN141), which encodes the major attachment protein of *M. pneumoniae*; in contrast to strain HAP157, HAP157FUP was found to have a deletion of an AGT triplet at position 182,792–182,794 within the P1 gene. This triplet is part of a previously described tandem repeat of which the biological relevance is yet unknown (Dorigo-Zetsma et al., 2001).

**Table 3:** **Variant frequencies of strains HAP157 and HAP157FUP**.

**SNV/InDels**	**Sequence ID**		**Locus**
**(NC_000912.1)**	**08**	**09**	
182792-182794:AGT-	0.66	0.01	MPN141
195459-195459:C-CA	0.25	0.04	NA
570767-570767:G-T	0.89	0.62	NA
570769-570769:A-G	0.88	0.61	NA
570770-570770:C-G	0.85	0.61	NA
622601-622601:G-GTT	0.04	0.40	NA
622601-622601:G-GT	0.41	0.14	NA
649041-649041:G-T	0.91	0.00	NA

*NA, Not applicable*.

### The sequences of isolates displaying differences in colony morphology

Two of the strains (C036-1 and C036-2) were isolated from the same patient at a single time point. These strains were analyzed separately because they displayed different colony morphologies on agar plates. The sequences of these strains were found to be highly similar (Figure 1): only 5 SNPs were identified between these strains (Table 4).

**Table 4:** **Variant frequencies of the isolates C036-1 and C036-2 with different colony morphologies**.

**SNV/InDels**	**Sequence ID**		**Locus**
**(NC_000912.1)**	**16**	**17**	
195459-195459:C-CA	0.03	0.25	NA
626378-626378:G-T	0.00	0.35	NA
649044-649044:G-C	0.99	0.00	NA
649047-649047:G-T	0.00	0.99	NA
716850-716850:C-A	0.00	0.38	MPN594

*NA, Not applicable*.

## Discussion

We determined the complete genome sequences of *M. pneumoniae* isolates obtained from asymptomatic *M. pneumoniae* carriers, and from patients suffering from an upper or lower RTI caused by *M. pneumoniae*. In addition, we analyzed the genomes of 3 *M. pneumoniae* reference strains. In a comparison of these sequences, we could not identify a specific genotype that is associated with *M. pneumoniae* virulence or asymptomatic bacterial carriage.

Our previous findings on sequence variation among *M. pneumoniae* strains, which mainly focussed on the P1 gene and repetitive elements (RepMP sequences), showed that variation among repetitive sequences is very common in this bacterium (Spuesens et al., 2009, 2010). In the current analysis of whole-genome sequences of 20 different *M. pneumoniae* isolates, however, we did not identify large genomic rearrangements. These findings are concordant with those of Xiao et al. In their analysis, the sequences of 15 *M. pneumoniae* strains seem very stable over time and at different locations in the world. Similarly, Lluch-Senar et al. (2015) found a relatively low number of non-synonymous SNPs among the genome sequences of 23 strains from 6 different countries. However, they do report a high rate of variation among repetitive elements in the *M. pneumoniae* genomes (Lluch-Senar et al., 2015).

Although we were able to reliably determine the genomic sequences of *M. pneumoniae* isolates from different well-defined groups of patients, the most important drawback of our study is the limited availability of *M. pneumoniae* isolates from asymptomatic patients. In routine microbiological diagnostics, asymptomatic patients are usually not tested for the presence of *M. pneumoniae*. In addition, in our previous study on asymptomatic carriage of *M. pneumoniae*, only a limited number of *M. pneumoniae* PCR-positive samples also turned out to be culture-positive (Spuesens et al., 2013). Clearly, for genomic sequence analysis, culturing is a crucial step in obtaining pure, clonal bacterial isolates. Nevertheless, in future studies, we aim to obtain higher number of *M. pneumoniae* isolates from different groups of asymptomatic individuals. This should provide more insight in the physiology of asymptomatic colonization of the human respiratory tract by *M. pneumoniae*.

Another important issue to consider is whether or not *M. pneumoniae* was the actual causative agent of the RTI in the different groups of symptomatic patients that were included in this study. Recent studies have indicated that multiple pathogens can be present in the respiratory tract of children and adults with RTI (Spuesens et al., 2013; Biesbroek et al., 2014a,b; Dickson et al., 2014). As a consequence, we cannot rule out that pathogens other than *M. pneumoniae* might have caused the symptomatic “phenotype” in at least some of the included symptomatic patients. Clearly, this may have obstructed the identification of potential genomic features that allow discrimination between pathogenic *M. pneumoniae* strains and strains that can be carried asymptomatically by the human host.

In conclusion, in this study we have shown that there is no specific genotype that can be associated with *M. pneumoniae* virulence or asymptomatic carriage. In addition, we found marked genetic differences between clinical isolates and the reference strains, which indicated that the latter strains may not be regarded as appropriate representatives of circulating *M. pneumoniae* strains.

## Author contributions

ES, AV, and CV initiated the study and were responsible for the original design. ES, RB, CK, KM, and TH all contributed to the execution of the study (specifically by retrieving patient clinical information, performance of the culture of the *M. pneumoniae* strains, DNA isolation and sequencing). RJ provided the clinical information and some of the *M. pneumoniae* strains. ES, RB, WV, AV, and CV analyzed and interpreted the data. ES wrote the first draft of the manuscript and was responsible for the subsequent modifications. All authors were involved in the final modifications of the manuscript.

### Conflict of interest statement

The authors declare that the research was conducted in the absence of any commercial or financial relationships that could be construed as a potential conflict of interest.

## Supplementary material

Supplementary File 1 **Fast QC files**. HTML files per strain. Each FastQC report includes: Basic Statistics, Per base sequence, quality, Per sequence quality scores, Per base sequence content, Per sequence GC content, Per base N content, Sequence Length Distribution, Sequence Duplication Levels, Overrepresented sequences, Adapter Content, and Kmer Content.

Supplementary File 2 **MUMmer alignments**. The alignments of the generated genome assemblies to the M129 reference genome.

Supplementary File 3 **Generated contigs**. Generated genome assemblies per strain.

Supplementary File 4 **Variants**.

Supplementary Figure 1 **The results of the optimization on the assembly statistics**.

Supplementary Table 1 **Differences between the reference sequence and the reads as determined by interpreting the output of samtools mpileup**.
