# Supplementary material for: Comparison of Mycoplasma pneumoniae Genome Sequences from Strains Isolated from Symptomatic and Asymptomatic Patients
Source: Front Microbiol. 2016 Oct 27;7:1701. doi: 10.3389/fmicb.2016.01701 (PMC5081376; doi:10.3389/fmicb.2016.01701)
Supplement: Supplementary File 1 — Fast QC files. HTML files per strain. Each FastQC report includes: Basic Statistics, Per base sequence, quality, Per sequence quality scores, Per base sequence content, Per sequence GC content, Per base N content, Sequence Length Distribution, Sequence Duplication Levels, Overrepresented sequences, Adapter Content, and Kmer Content. [file DataSheet1.zip › Supplementary files/Supplementary file 1 FastQC/I12-1149-01_interleaved_fastqc.html]

I12-1149-01\_interleaved.fastq FastQC Report 

FastQC Report

Mon 4 Jul 2016  
I12-1149-01\_interleaved.fastq

## Summary

- Basic Statistics
- Per base sequence quality
- Per sequence quality scores
- Per base sequence content
- Per sequence GC content
- Per base N content
- Sequence Length Distribution
- Sequence Duplication Levels
- Overrepresented sequences
- Adapter Content
- Kmer Content

## Basic Statistics

| Measure | Value |
| --- | --- |
| Filename | I12-1149-01\_interleaved.fastq |
| File type | Conventional base calls |
| Encoding | Sanger / Illumina 1.9 |
| Total Sequences | 12312986 |
| Sequences flagged as poor quality | 0 |
| Sequence length | 101 |
| %GC | 39 |

## Per base sequence quality

## Per sequence quality scores

## Per base sequence content

## Per sequence GC content

## Per base N content

## Sequence Length Distribution

## Sequence Duplication Levels

## Overrepresented sequences

No overrepresented sequences

## Adapter Content

## Kmer Content

| Sequence | Count | PValue | Obs/Exp Max | Max Obs/Exp Position |
| --- | --- | --- | --- | --- |
| GGCGCCG | 910 | 0.0 | 11.497375 | 44-45 |
| GTCGCCG | 4465 | 0.0 | 10.7576475 | 44-45 |
| CCGTATC | 5100 | 0.0 | 9.967095 | 48-49 |
| TCTCGGG | 855 | 0.0 | 9.727614 | 36-37 |
| CGCCGTA | 5380 | 0.0 | 9.326211 | 46-47 |
| GTATCAT | 6280 | 0.0 | 8.460985 | 50-51 |
| TCGGGGG | 2570 | 0.0 | 7.584093 | 38-39 |
| GCGTCGG | 1125 | 0.0 | 7.39523 | 94-95 |
| GCCGTAT | 5015 | 0.0 | 7.246787 | 48-49 |
| GAGCGGC | 1550 | 2.4756446E-9 | 7.04709 | 9 |
| GGGCGCC | 1510 | 0.0 | 6.7713103 | 42-43 |
| GGGCCCG | 565 | 1.2076813E-5 | 6.73378 | 44-45 |
| CGTCGGG | 1045 | 1.8189894E-11 | 6.596418 | 12-13 |
| CGTATCA | 5900 | 0.0 | 6.593627 | 50-51 |
| CGCCGGA | 1605 | 0.0 | 6.519026 | 46-47 |
| GGTCGCC | 5180 | 0.0 | 6.4266653 | 44-45 |
| GATCTCG | 9815 | 0.0 | 6.1707854 | 34-35 |
| TGGTCGC | 7620 | 0.0 | 6.022593 | 42-43 |
| TCTCGGT | 9140 | 0.0 | 5.953793 | 36-37 |
| ATATAGG | 1545 | 5.53857E-6 | 5.832851 | 2 |

Produced by FastQC (version 0.11.5)
