# Supplementary material for: Comparison of Mycoplasma pneumoniae Genome Sequences from Strains Isolated from Symptomatic and Asymptomatic Patients
Source: Front Microbiol. 2016 Oct 27;7:1701. doi: 10.3389/fmicb.2016.01701 (PMC5081376; doi:10.3389/fmicb.2016.01701)
Supplement: Supplementary File 1 — Fast QC files. HTML files per strain. Each FastQC report includes: Basic Statistics, Per base sequence, quality, Per sequence quality scores, Per base sequence content, Per sequence GC content, Per base N content, Sequence Length Distribution, Sequence Duplication Levels, Overrepresented sequences, Adapter Content, and Kmer Content. [file DataSheet1.zip › Supplementary files/Supplementary file 1 FastQC/I12-1149-02_interleaved_fastqc.html]

I12-1149-02\_interleaved.fastq FastQC Report 

FastQC Report

Mon 4 Jul 2016  
I12-1149-02\_interleaved.fastq

## Summary

- Basic Statistics
- Per base sequence quality
- Per sequence quality scores
- Per base sequence content
- Per sequence GC content
- Per base N content
- Sequence Length Distribution
- Sequence Duplication Levels
- Overrepresented sequences
- Adapter Content
- Kmer Content

## Basic Statistics

| Measure | Value |
| --- | --- |
| Filename | I12-1149-02\_interleaved.fastq |
| File type | Conventional base calls |
| Encoding | Sanger / Illumina 1.9 |
| Total Sequences | 15880668 |
| Sequences flagged as poor quality | 0 |
| Sequence length | 101 |
| %GC | 39 |

## Per base sequence quality

## Per sequence quality scores

## Per base sequence content

## Per sequence GC content

## Per base N content

## Sequence Length Distribution

## Sequence Duplication Levels

## Overrepresented sequences

| Sequence | Count | Percentage | Possible Source |
| --- | --- | --- | --- |
| GATCGGAAGAGCACACGTCTGAACTCCAGTCACCGATGTATCTCGTATGC | 65209 | 0.41061874727184017 | TruSeq Adapter, Index 2 (100% over 50bp) |
| AGATCGGAAGAGCGTCGTGTAGGGAAAGAGTGTAGATCTCGGTGGTCGCC | 38214 | 0.24063219506887243 | Illumina Single End PCR Primer 1 (100% over 50bp) |
| AGATCGGAAGAGCACACGTCTGAACTCCAGTCACCGATGTATCTCGTATG | 37480 | 0.23601022324753596 | TruSeq Adapter, Index 2 (100% over 49bp) |
| GATCGGAAGAGCGTCGTGTAGGGAAAGAGTGTAGATCTCGGTGGTCGCCG | 23696 | 0.14921286686429058 | Illumina Single End PCR Primer 1 (100% over 50bp) |

## Adapter Content

## Kmer Content

| Sequence | Count | PValue | Obs/Exp Max | Max Obs/Exp Position |
| --- | --- | --- | --- | --- |
| GAGCGGC | 3665 | 0.0 | 39.26219 | 9 |
| GAGGGGC | 2235 | 0.0 | 35.697487 | 9 |
| CGGGAGA | 2860 | 0.0 | 33.823715 | 4 |
| GGCGCCG | 4825 | 0.0 | 33.068916 | 44-45 |
| AGAGCGG | 4895 | 0.0 | 31.897861 | 8 |
| TCTCGGG | 4565 | 0.0 | 30.140236 | 36-37 |
| GAGAGGG | 3055 | 0.0 | 29.499672 | 7 |
| GGGAGAG | 4410 | 0.0 | 28.709236 | 5 |
| TCGGGGG | 9130 | 0.0 | 28.429352 | 38-39 |
| GGGCGCC | 6515 | 0.0 | 27.921562 | 42-43 |
| CCGTATC | 30460 | 0.0 | 27.89352 | 48-49 |
| GTATCAT | 30630 | 0.0 | 27.538717 | 50-51 |
| CGCCGTA | 31310 | 0.0 | 26.756912 | 46-47 |
| TCGGGAG | 3400 | 0.0 | 25.385069 | 3 |
| GTCGCCG | 31320 | 0.0 | 25.373444 | 44-45 |
| GATCGGG | 4995 | 0.0 | 25.361347 | 1 |
| CGCCGGA | 4140 | 0.0 | 24.411531 | 46-47 |
| GGAGAGC | 2780 | 0.0 | 24.391977 | 6 |
| CGTCGGG | 3025 | 0.0 | 24.359394 | 12-13 |
| GGAGAGG | 3815 | 0.0 | 24.237947 | 6 |

Produced by FastQC (version 0.11.5)
