# Supplementary material for: Comparison of Mycoplasma pneumoniae Genome Sequences from Strains Isolated from Symptomatic and Asymptomatic Patients
Source: Front Microbiol. 2016 Oct 27;7:1701. doi: 10.3389/fmicb.2016.01701 (PMC5081376; doi:10.3389/fmicb.2016.01701)
Supplement: Supplementary File 1 — Fast QC files. HTML files per strain. Each FastQC report includes: Basic Statistics, Per base sequence, quality, Per sequence quality scores, Per base sequence content, Per sequence GC content, Per base N content, Sequence Length Distribution, Sequence Duplication Levels, Overrepresented sequences, Adapter Content, and Kmer Content. [file DataSheet1.zip › Supplementary files/Supplementary file 1 FastQC/I12-1149-03_interleaved_fastqc.html]

I12-1149-03\_interleaved.fastq FastQC Report 

FastQC Report

Mon 4 Jul 2016  
I12-1149-03\_interleaved.fastq

## Summary

- Basic Statistics
- Per base sequence quality
- Per sequence quality scores
- Per base sequence content
- Per sequence GC content
- Per base N content
- Sequence Length Distribution
- Sequence Duplication Levels
- Overrepresented sequences
- Adapter Content
- Kmer Content

## Basic Statistics

| Measure | Value |
| --- | --- |
| Filename | I12-1149-03\_interleaved.fastq |
| File type | Conventional base calls |
| Encoding | Sanger / Illumina 1.9 |
| Total Sequences | 18237272 |
| Sequences flagged as poor quality | 0 |
| Sequence length | 101 |
| %GC | 40 |

## Per base sequence quality

## Per sequence quality scores

## Per base sequence content

## Per sequence GC content

## Per base N content

## Sequence Length Distribution

## Sequence Duplication Levels

## Overrepresented sequences

| Sequence | Count | Percentage | Possible Source |
| --- | --- | --- | --- |
| GATCGGAAGAGCACACGTCTGAACTCCAGTCACTTAGGCATCTCGTATGC | 55049 | 0.3018488730112705 | TruSeq Adapter, Index 3 (100% over 50bp) |
| GATCGGAAGAGCGTCGTGTAGGGAAAGAGTGTAGATCTCGGTGGTCGCCG | 20919 | 0.11470465538924901 | Illumina Single End PCR Primer 1 (100% over 50bp) |

## Adapter Content

## Kmer Content

| Sequence | Count | PValue | Obs/Exp Max | Max Obs/Exp Position |
| --- | --- | --- | --- | --- |
| GTCGCCG | 12085 | 0.0 | 32.956398 | 44-45 |
| CGCCGTA | 13320 | 0.0 | 30.883207 | 46-47 |
| GAGCGGC | 3625 | 0.0 | 30.393574 | 9 |
| GGCGCCG | 3685 | 0.0 | 30.06907 | 44-45 |
| TCTCGGG | 3110 | 0.0 | 30.027277 | 36-37 |
| CCGTATC | 13925 | 0.0 | 29.970741 | 48-49 |
| GAGGGGC | 2435 | 0.0 | 28.474474 | 9 |
| GAGAGGG | 2835 | 0.0 | 28.276865 | 7 |
| GGGCGCC | 5315 | 0.0 | 27.289396 | 42-43 |
| GTATCAT | 15335 | 0.0 | 26.79401 | 50-51 |
| CGGGAGA | 3155 | 0.0 | 26.60397 | 4 |
| GATCTCG | 19305 | 0.0 | 25.405354 | 34-35 |
| TCGGGGG | 8095 | 0.0 | 24.429426 | 38-39 |
| AGAGCGG | 4915 | 0.0 | 24.238535 | 8 |
| GGTCGCC | 12995 | 0.0 | 24.17096 | 42-43 |
| GGGAGAG | 4740 | 0.0 | 23.809664 | 5 |
| GCCGTAT | 13715 | 0.0 | 23.197481 | 46-47 |
| TGGTCGC | 15960 | 0.0 | 22.451624 | 42-43 |
| TCTCGGT | 17835 | 0.0 | 22.449703 | 36-37 |
| ATCTCGG | 18020 | 0.0 | 21.81314 | 34-35 |

Produced by FastQC (version 0.11.5)
