# Supplementary material for: Comparison of Mycoplasma pneumoniae Genome Sequences from Strains Isolated from Symptomatic and Asymptomatic Patients
Source: Front Microbiol. 2016 Oct 27;7:1701. doi: 10.3389/fmicb.2016.01701 (PMC5081376; doi:10.3389/fmicb.2016.01701)
Supplement: Supplementary File 1 — Fast QC files. HTML files per strain. Each FastQC report includes: Basic Statistics, Per base sequence, quality, Per sequence quality scores, Per base sequence content, Per sequence GC content, Per base N content, Sequence Length Distribution, Sequence Duplication Levels, Overrepresented sequences, Adapter Content, and Kmer Content. [file DataSheet1.zip › Supplementary files/Supplementary file 1 FastQC/I12-1149-04_interleaved_fastqc.html]

I12-1149-04\_interleaved.fastq FastQC Report 

FastQC Report

Mon 4 Jul 2016  
I12-1149-04\_interleaved.fastq

## Summary

- Basic Statistics
- Per base sequence quality
- Per sequence quality scores
- Per base sequence content
- Per sequence GC content
- Per base N content
- Sequence Length Distribution
- Sequence Duplication Levels
- Overrepresented sequences
- Adapter Content
- Kmer Content

## Basic Statistics

| Measure | Value |
| --- | --- |
| Filename | I12-1149-04\_interleaved.fastq |
| File type | Conventional base calls |
| Encoding | Sanger / Illumina 1.9 |
| Total Sequences | 22931298 |
| Sequences flagged as poor quality | 0 |
| Sequence length | 101 |
| %GC | 40 |

## Per base sequence quality

## Per sequence quality scores

## Per base sequence content

## Per sequence GC content

## Per base N content

## Sequence Length Distribution

## Sequence Duplication Levels

## Overrepresented sequences

| Sequence | Count | Percentage | Possible Source |
| --- | --- | --- | --- |
| GATCGGAAGAGCACACGTCTGAACTCCAGTCACTGACCAATCTCGTATGC | 46719 | 0.20373465121773746 | TruSeq Adapter, Index 4 (100% over 50bp) |

## Adapter Content

## Kmer Content

| Sequence | Count | PValue | Obs/Exp Max | Max Obs/Exp Position |
| --- | --- | --- | --- | --- |
| GTCGCCG | 11770 | 0.0 | 33.69743 | 44-45 |
| CGCCGTA | 13750 | 0.0 | 29.77955 | 46-47 |
| CCGTATC | 14475 | 0.0 | 28.750288 | 48-49 |
| TCTCGGG | 3495 | 0.0 | 28.420635 | 36-37 |
| GGCGCCG | 3840 | 0.0 | 27.307663 | 44-45 |
| GAGCGGC | 4215 | 0.0 | 27.04056 | 9 |
| GGGCGCC | 5450 | 0.0 | 25.61046 | 42-43 |
| GATCTCG | 19465 | 0.0 | 25.416931 | 34-35 |
| GTATCAT | 16550 | 0.0 | 25.199411 | 50-51 |
| GGTCGCC | 12610 | 0.0 | 23.85345 | 42-43 |
| CGGGAGA | 3520 | 0.0 | 23.305937 | 4 |
| GAGAGGG | 3175 | 0.0 | 23.156218 | 7 |
| GAGGGGC | 3005 | 0.0 | 22.125116 | 9 |
| TCTCGGT | 18505 | 0.0 | 21.83054 | 36-37 |
| GCCGTAT | 14070 | 0.0 | 21.733742 | 46-47 |
| ATCTCGG | 17865 | 0.0 | 21.644106 | 34-35 |
| TGGTCGC | 17035 | 0.0 | 21.342297 | 42-43 |
| TCGGGGG | 9395 | 0.0 | 20.772316 | 38-39 |
| TAGATCT | 22360 | 0.0 | 20.54791 | 32-33 |
| GGGAGAG | 5215 | 0.0 | 20.276936 | 5 |

Produced by FastQC (version 0.11.5)
