# Supplementary material for: Comparison of Mycoplasma pneumoniae Genome Sequences from Strains Isolated from Symptomatic and Asymptomatic Patients
Source: Front Microbiol. 2016 Oct 27;7:1701. doi: 10.3389/fmicb.2016.01701 (PMC5081376; doi:10.3389/fmicb.2016.01701)
Supplement: Supplementary File 1 — Fast QC files. HTML files per strain. Each FastQC report includes: Basic Statistics, Per base sequence, quality, Per sequence quality scores, Per base sequence content, Per sequence GC content, Per base N content, Sequence Length Distribution, Sequence Duplication Levels, Overrepresented sequences, Adapter Content, and Kmer Content. [file DataSheet1.zip › Supplementary files/Supplementary file 1 FastQC/I12-1149-05_interleaved_fastqc.html]

I12-1149-05\_interleaved.fastq FastQC Report 

FastQC Report

Mon 4 Jul 2016  
I12-1149-05\_interleaved.fastq

## Summary

- Basic Statistics
- Per base sequence quality
- Per sequence quality scores
- Per base sequence content
- Per sequence GC content
- Per base N content
- Sequence Length Distribution
- Sequence Duplication Levels
- Overrepresented sequences
- Adapter Content
- Kmer Content

## Basic Statistics

| Measure | Value |
| --- | --- |
| Filename | I12-1149-05\_interleaved.fastq |
| File type | Conventional base calls |
| Encoding | Sanger / Illumina 1.9 |
| Total Sequences | 24359148 |
| Sequences flagged as poor quality | 0 |
| Sequence length | 101 |
| %GC | 39 |

## Per base sequence quality

## Per sequence quality scores

## Per base sequence content

## Per sequence GC content

## Per base N content

## Sequence Length Distribution

## Sequence Duplication Levels

## Overrepresented sequences

| Sequence | Count | Percentage | Possible Source |
| --- | --- | --- | --- |
| GATCGGAAGAGCACACGTCTGAACTCCAGTCACACAGTGATCTCGTATGC | 47602 | 0.1954173438249975 | TruSeq Adapter, Index 5 (100% over 50bp) |

## Adapter Content

## Kmer Content

| Sequence | Count | PValue | Obs/Exp Max | Max Obs/Exp Position |
| --- | --- | --- | --- | --- |
| TCTCGGG | 3470 | 0.0 | 28.761103 | 36-37 |
| GGCGCCG | 3915 | 0.0 | 26.90777 | 44-45 |
| GTCGCCG | 12270 | 0.0 | 26.841759 | 44-45 |
| GAGCGGC | 4205 | 0.0 | 25.861904 | 9 |
| GGGCGCC | 5765 | 0.0 | 25.037457 | 42-43 |
| CGCCGTA | 13810 | 0.0 | 24.607046 | 46-47 |
| CGGGAGA | 3505 | 0.0 | 24.488256 | 4 |
| CCGTATC | 14270 | 0.0 | 24.268919 | 48-49 |
| GAGAGGG | 3515 | 0.0 | 21.725443 | 7 |
| GAGGGGC | 2910 | 0.0 | 21.541304 | 9 |
| GGTCGCC | 13505 | 0.0 | 21.534397 | 42-43 |
| GTATCAT | 16170 | 0.0 | 21.28563 | 50-51 |
| GCCGTAT | 13900 | 0.0 | 21.19715 | 46-47 |
| TCGGGGG | 9350 | 0.0 | 20.79508 | 38-39 |
| GGGAGAG | 5315 | 0.0 | 20.787537 | 5 |
| AGAGCGG | 5705 | 0.0 | 20.546833 | 8 |
| CGTCGGG | 2980 | 0.0 | 20.180403 | 12-13 |
| GATCTCG | 21165 | 0.0 | 19.559212 | 34-35 |
| GCGTCGG | 2925 | 0.0 | 19.491774 | 10-11 |
| ATCTCGG | 19290 | 0.0 | 19.108725 | 34-35 |

Produced by FastQC (version 0.11.5)
