# Supplementary material for: Comparison of Mycoplasma pneumoniae Genome Sequences from Strains Isolated from Symptomatic and Asymptomatic Patients
Source: Front Microbiol. 2016 Oct 27;7:1701. doi: 10.3389/fmicb.2016.01701 (PMC5081376; doi:10.3389/fmicb.2016.01701)
Supplement: Supplementary File 1 — Fast QC files. HTML files per strain. Each FastQC report includes: Basic Statistics, Per base sequence, quality, Per sequence quality scores, Per base sequence content, Per sequence GC content, Per base N content, Sequence Length Distribution, Sequence Duplication Levels, Overrepresented sequences, Adapter Content, and Kmer Content. [file DataSheet1.zip › Supplementary files/Supplementary file 1 FastQC/I12-1149-06_interleaved_fastqc.html]

I12-1149-06\_interleaved.fastq FastQC Report 

FastQC Report

Mon 4 Jul 2016  
I12-1149-06\_interleaved.fastq

## Summary

- Basic Statistics
- Per base sequence quality
- Per sequence quality scores
- Per base sequence content
- Per sequence GC content
- Per base N content
- Sequence Length Distribution
- Sequence Duplication Levels
- Overrepresented sequences
- Adapter Content
- Kmer Content

## Basic Statistics

| Measure | Value |
| --- | --- |
| Filename | I12-1149-06\_interleaved.fastq |
| File type | Conventional base calls |
| Encoding | Sanger / Illumina 1.9 |
| Total Sequences | 14236426 |
| Sequences flagged as poor quality | 0 |
| Sequence length | 101 |
| %GC | 39 |

## Per base sequence quality

## Per sequence quality scores

## Per base sequence content

## Per sequence GC content

## Per base N content

## Sequence Length Distribution

## Sequence Duplication Levels

## Overrepresented sequences

| Sequence | Count | Percentage | Possible Source |
| --- | --- | --- | --- |
| GATCGGAAGAGCACACGTCTGAACTCCAGTCACCAGATCATCTCGTATGC | 194768 | 1.3680961780716592 | TruSeq Adapter, Index 7 (100% over 50bp) |
| GATCGGAAGAGCGTCGTGTAGGGAAAGAGTGTAGATCTCGGTGGTCGCCG | 41339 | 0.29037484548439335 | Illumina Single End PCR Primer 1 (100% over 50bp) |
| AGATCGGAAGAGCGTCGTGTAGGGAAAGAGTGTAGATCTCGGTGGTCGCC | 40877 | 0.2871296489722912 | Illumina Single End PCR Primer 1 (100% over 50bp) |
| AGATCGGAAGAGCACACGTCTGAACTCCAGTCACCAGATCATCTCGTATG | 39065 | 0.2744017353793712 | TruSeq Adapter, Index 7 (100% over 49bp) |

## Adapter Content

## Kmer Content

| Sequence | Count | PValue | Obs/Exp Max | Max Obs/Exp Position |
| --- | --- | --- | --- | --- |
| GAGCGGC | 6660 | 0.0 | 66.244804 | 9 |
| CGGGAGA | 5255 | 0.0 | 61.809444 | 4 |
| AGAGCGG | 8360 | 0.0 | 56.77054 | 8 |
| GAGGGGC | 4470 | 0.0 | 55.88414 | 9 |
| GATCGGG | 7490 | 0.0 | 54.41268 | 1 |
| TCGGGAG | 5535 | 0.0 | 53.204964 | 3 |
| GAGAGCG | 4245 | 0.0 | 47.8202 | 7 |
| GGAGAGC | 4465 | 0.0 | 47.785316 | 6 |
| GAGAGGG | 5860 | 0.0 | 47.59102 | 7 |
| GGGAGAG | 8705 | 0.0 | 46.623222 | 5 |
| GGAGAGG | 6520 | 0.0 | 44.432117 | 6 |
| CGGAGAG | 1955 | 0.0 | 43.168858 | 5 |
| ATCGGGA | 9465 | 0.0 | 42.946114 | 2 |
| GGCGCCG | 9945 | 0.0 | 41.556244 | 44-45 |
| TCTCGGG | 9075 | 0.0 | 41.29555 | 36-37 |
| GAGGGTC | 2625 | 0.0 | 39.98281 | 9 |
| AAGGGGC | 7100 | 0.0 | 39.464264 | 9 |
| CCGGAGA | 1310 | 0.0 | 38.73017 | 4 |
| TCGGGGG | 20235 | 0.0 | 38.529045 | 38-39 |
| GGGCGCC | 14330 | 0.0 | 37.153126 | 42-43 |

Produced by FastQC (version 0.11.5)
