# Supplementary material for: Comparison of Mycoplasma pneumoniae Genome Sequences from Strains Isolated from Symptomatic and Asymptomatic Patients
Source: Front Microbiol. 2016 Oct 27;7:1701. doi: 10.3389/fmicb.2016.01701 (PMC5081376; doi:10.3389/fmicb.2016.01701)
Supplement: Supplementary File 1 — Fast QC files. HTML files per strain. Each FastQC report includes: Basic Statistics, Per base sequence, quality, Per sequence quality scores, Per base sequence content, Per sequence GC content, Per base N content, Sequence Length Distribution, Sequence Duplication Levels, Overrepresented sequences, Adapter Content, and Kmer Content. [file DataSheet1.zip › Supplementary files/Supplementary file 1 FastQC/I12-1149-07_interleaved_fastqc.html]

I12-1149-07\_interleaved.fastq FastQC Report 

FastQC Report

Mon 4 Jul 2016  
I12-1149-07\_interleaved.fastq

## Summary

- Basic Statistics
- Per base sequence quality
- Per sequence quality scores
- Per base sequence content
- Per sequence GC content
- Per base N content
- Sequence Length Distribution
- Sequence Duplication Levels
- Overrepresented sequences
- Adapter Content
- Kmer Content

## Basic Statistics

| Measure | Value |
| --- | --- |
| Filename | I12-1149-07\_interleaved.fastq |
| File type | Conventional base calls |
| Encoding | Sanger / Illumina 1.9 |
| Total Sequences | 26409770 |
| Sequences flagged as poor quality | 0 |
| Sequence length | 101 |
| %GC | 38 |

## Per base sequence quality

## Per sequence quality scores

## Per base sequence content

## Per sequence GC content

## Per base N content

## Sequence Length Distribution

## Sequence Duplication Levels

## Overrepresented sequences

No overrepresented sequences

## Adapter Content

## Kmer Content

| Sequence | Count | PValue | Obs/Exp Max | Max Obs/Exp Position |
| --- | --- | --- | --- | --- |
| CCGTATC | 12420 | 0.0 | 13.578431 | 48-49 |
| GTCGCCG | 12000 | 0.0 | 13.296522 | 44-45 |
| CGCCGTA | 12675 | 0.0 | 13.001603 | 46-47 |
| GGCGCCG | 1940 | 0.0 | 12.134733 | 44-45 |
| GTATCAT | 14420 | 0.0 | 11.794549 | 50-51 |
| TCTCGGG | 1915 | 0.0 | 11.043993 | 36-37 |
| GCGTCGG | 2615 | 0.0 | 9.817805 | 94-95 |
| GGGCGCC | 2900 | 0.0 | 9.265626 | 42-43 |
| TCGGGGG | 5120 | 0.0 | 8.77436 | 38-39 |
| GGGGCCC | 1645 | 0.0 | 8.673255 | 44-45 |
| GCCGTAT | 12375 | 0.0 | 8.560567 | 48-49 |
| TGGTCGC | 18575 | 0.0 | 8.052229 | 42-43 |
| CGTATCA | 13745 | 0.0 | 8.007568 | 50-51 |
| GGGGGCC | 2300 | 0.0 | 7.8574214 | 42-43 |
| GGTCGCC | 13355 | 0.0 | 7.7631745 | 44-45 |
| GGGCCCG | 900 | 0.0 | 7.6621785 | 44-45 |
| GATCTCG | 25790 | 0.0 | 7.284485 | 34-35 |
| GAGCGGC | 3325 | 0.0 | 7.141256 | 9 |
| TCGCCGT | 14105 | 0.0 | 7.080889 | 46-47 |
| TCGGTGG | 25205 | 0.0 | 6.9880357 | 38-39 |

Produced by FastQC (version 0.11.5)
