# Supplementary material for: Comparison of Mycoplasma pneumoniae Genome Sequences from Strains Isolated from Symptomatic and Asymptomatic Patients
Source: Front Microbiol. 2016 Oct 27;7:1701. doi: 10.3389/fmicb.2016.01701 (PMC5081376; doi:10.3389/fmicb.2016.01701)
Supplement: Supplementary File 1 — Fast QC files. HTML files per strain. Each FastQC report includes: Basic Statistics, Per base sequence, quality, Per sequence quality scores, Per base sequence content, Per sequence GC content, Per base N content, Sequence Length Distribution, Sequence Duplication Levels, Overrepresented sequences, Adapter Content, and Kmer Content. [file DataSheet1.zip › Supplementary files/Supplementary file 1 FastQC/I12-1149-08_interleaved_fastqc.html]

I12-1149-08\_interleaved.fastq FastQC Report 

FastQC Report

Mon 4 Jul 2016  
I12-1149-08\_interleaved.fastq

## Summary

- Basic Statistics
- Per base sequence quality
- Per sequence quality scores
- Per base sequence content
- Per sequence GC content
- Per base N content
- Sequence Length Distribution
- Sequence Duplication Levels
- Overrepresented sequences
- Adapter Content
- Kmer Content

## Basic Statistics

| Measure | Value |
| --- | --- |
| Filename | I12-1149-08\_interleaved.fastq |
| File type | Conventional base calls |
| Encoding | Sanger / Illumina 1.9 |
| Total Sequences | 18976996 |
| Sequences flagged as poor quality | 0 |
| Sequence length | 101 |
| %GC | 40 |

## Per base sequence quality

## Per sequence quality scores

## Per base sequence content

## Per sequence GC content

## Per base N content

## Sequence Length Distribution

## Sequence Duplication Levels

## Overrepresented sequences

No overrepresented sequences

## Adapter Content

## Kmer Content

| Sequence | Count | PValue | Obs/Exp Max | Max Obs/Exp Position |
| --- | --- | --- | --- | --- |
| GTCGCCG | 3100 | 0.0 | 18.638493 | 44-45 |
| CGCCGTA | 4745 | 0.0 | 12.377902 | 46-47 |
| GGCGCCG | 1390 | 0.0 | 12.316403 | 44-45 |
| CCGTATC | 5165 | 0.0 | 11.727355 | 48-49 |
| GGTCGCC | 4110 | 0.0 | 11.339104 | 42-43 |
| TCTCGGG | 1290 | 0.0 | 11.237017 | 36-37 |
| GGGCGCC | 2110 | 0.0 | 10.254711 | 42-43 |
| GATCTCG | 7855 | 0.0 | 9.313501 | 34-35 |
| ATCTCGG | 6180 | 0.0 | 9.185819 | 34-35 |
| GCCGTAT | 5055 | 0.0 | 9.031635 | 46-47 |
| GTATCAT | 6995 | 0.0 | 8.851267 | 50-51 |
| GAGCGGC | 2630 | 0.0 | 7.5844216 | 9 |
| TCTCGGT | 8505 | 0.0 | 7.516036 | 36-37 |
| TCGCCGT | 5595 | 0.0 | 7.2671185 | 44-45 |
| TGGTCGC | 7920 | 0.0 | 7.265319 | 42-43 |
| CGTATCA | 6410 | 0.0 | 7.1890903 | 48-49 |
| TCGGGGG | 4490 | 0.0 | 6.93502 | 38-39 |
| TCGGTGG | 10545 | 0.0 | 6.6496406 | 38-39 |
| GGAGAGC | 2365 | 1.0913936E-11 | 6.215477 | 6 |
| TAGATCT | 11220 | 0.0 | 6.1942134 | 32-33 |

Produced by FastQC (version 0.11.5)
