# Supplementary material for: Comparison of Mycoplasma pneumoniae Genome Sequences from Strains Isolated from Symptomatic and Asymptomatic Patients
Source: Front Microbiol. 2016 Oct 27;7:1701. doi: 10.3389/fmicb.2016.01701 (PMC5081376; doi:10.3389/fmicb.2016.01701)
Supplement: Supplementary File 1 — Fast QC files. HTML files per strain. Each FastQC report includes: Basic Statistics, Per base sequence, quality, Per sequence quality scores, Per base sequence content, Per sequence GC content, Per base N content, Sequence Length Distribution, Sequence Duplication Levels, Overrepresented sequences, Adapter Content, and Kmer Content. [file DataSheet1.zip › Supplementary files/Supplementary file 1 FastQC/I12-1149-09_interleaved_fastqc.html]

I12-1149-09\_interleaved.fastq FastQC Report 

FastQC Report

Mon 4 Jul 2016  
I12-1149-09\_interleaved.fastq

## Summary

- Basic Statistics
- Per base sequence quality
- Per sequence quality scores
- Per base sequence content
- Per sequence GC content
- Per base N content
- Sequence Length Distribution
- Sequence Duplication Levels
- Overrepresented sequences
- Adapter Content
- Kmer Content

## Basic Statistics

| Measure | Value |
| --- | --- |
| Filename | I12-1149-09\_interleaved.fastq |
| File type | Conventional base calls |
| Encoding | Sanger / Illumina 1.9 |
| Total Sequences | 17166632 |
| Sequences flagged as poor quality | 0 |
| Sequence length | 101 |
| %GC | 39 |

## Per base sequence quality

## Per sequence quality scores

## Per base sequence content

## Per sequence GC content

## Per base N content

## Sequence Length Distribution

## Sequence Duplication Levels

## Overrepresented sequences

| Sequence | Count | Percentage | Possible Source |
| --- | --- | --- | --- |
| GATCGGAAGAGCACACGTCTGAACTCCAGTCACGTTTCGGAATCTCGTAT | 328026 | 1.9108349267346094 | TruSeq Adapter, Index 21 (97% over 40bp) |
| GATCGGAAGAGCGTCGTGTAGGGAAAGAGTGTAGATCTCGGTGGTCGCCG | 98344 | 0.5728788267844269 | Illumina Single End PCR Primer 1 (100% over 50bp) |
| AGATCGGAAGAGCGTCGTGTAGGGAAAGAGTGTAGATCTCGGTGGTCGCC | 58353 | 0.3399210747920734 | Illumina Single End PCR Primer 1 (100% over 50bp) |
| AGATCGGAAGAGCACACGTCTGAACTCCAGTCACGTTTCGGAATCTCGTA | 54660 | 0.3184084099897988 | TruSeq Adapter, Index 21 (97% over 40bp) |
| GATCGGAGGAGCACACGTCTGAACTCCAGTCACGTTTCGGAATCTCGTAT | 22484 | 0.13097502177480125 | TruSeq Adapter, Index 21 (97% over 40bp) |

## Adapter Content

## Kmer Content

| Sequence | Count | PValue | Obs/Exp Max | Max Obs/Exp Position |
| --- | --- | --- | --- | --- |
| GAGCGGC | 10970 | 0.0 | 69.480034 | 9 |
| CGGGAGA | 7700 | 0.0 | 64.66413 | 4 |
| AGAGCGG | 12635 | 0.0 | 64.11316 | 8 |
| GAGGGGC | 6380 | 0.0 | 61.333557 | 9 |
| GATCGGG | 10765 | 0.0 | 59.32498 | 1 |
| TCGGGAG | 8150 | 0.0 | 55.919044 | 3 |
| GAGAGCG | 6035 | 0.0 | 55.87944 | 7 |
| GGAGAGC | 6530 | 0.0 | 54.027138 | 6 |
| ATCGGGA | 12680 | 0.0 | 49.337276 | 2 |
| GGGAGAG | 13525 | 0.0 | 48.69797 | 5 |
| GAGAGGG | 9395 | 0.0 | 47.65791 | 7 |
| AAGGGGC | 9915 | 0.0 | 47.129623 | 9 |
| GGAGAGG | 10130 | 0.0 | 45.12527 | 6 |
| CGGAGAG | 2940 | 0.0 | 44.99899 | 5 |
| CCGGAGA | 1820 | 0.0 | 44.81485 | 4 |
| GAGGGTC | 3830 | 0.0 | 43.025143 | 9 |
| TCTCGGG | 14425 | 0.0 | 41.726788 | 36-37 |
| GGCGCCG | 16680 | 0.0 | 40.85781 | 44-45 |
| TCGGGGG | 32065 | 0.0 | 39.392136 | 38-39 |
| GGGCGTC | 1810 | 0.0 | 38.830654 | 9 |

Produced by FastQC (version 0.11.5)
