# Supplementary material for: Comparison of Mycoplasma pneumoniae Genome Sequences from Strains Isolated from Symptomatic and Asymptomatic Patients
Source: Front Microbiol. 2016 Oct 27;7:1701. doi: 10.3389/fmicb.2016.01701 (PMC5081376; doi:10.3389/fmicb.2016.01701)
Supplement: Supplementary File 1 — Fast QC files. HTML files per strain. Each FastQC report includes: Basic Statistics, Per base sequence, quality, Per sequence quality scores, Per base sequence content, Per sequence GC content, Per base N content, Sequence Length Distribution, Sequence Duplication Levels, Overrepresented sequences, Adapter Content, and Kmer Content. [file DataSheet1.zip › Supplementary files/Supplementary file 1 FastQC/I12-1149-10_interleaved_fastqc.html]

I12-1149-10\_interleaved.fastq FastQC Report 

FastQC Report

Mon 4 Jul 2016  
I12-1149-10\_interleaved.fastq

## Summary

- Basic Statistics
- Per base sequence quality
- Per sequence quality scores
- Per base sequence content
- Per sequence GC content
- Per base N content
- Sequence Length Distribution
- Sequence Duplication Levels
- Overrepresented sequences
- Adapter Content
- Kmer Content

## Basic Statistics

| Measure | Value |
| --- | --- |
| Filename | I12-1149-10\_interleaved.fastq |
| File type | Conventional base calls |
| Encoding | Sanger / Illumina 1.9 |
| Total Sequences | 9562958 |
| Sequences flagged as poor quality | 0 |
| Sequence length | 101 |
| %GC | 39 |

## Per base sequence quality

## Per sequence quality scores

## Per base sequence content

## Per sequence GC content

## Per base N content

## Sequence Length Distribution

## Sequence Duplication Levels

## Overrepresented sequences

| Sequence | Count | Percentage | Possible Source |
| --- | --- | --- | --- |
| GATCGGAAGAGCACACGTCTGAACTCCAGTCACGATCAGATCTCGTATGC | 19154 | 0.20029367482320848 | TruSeq Adapter, Index 9 (100% over 50bp) |

## Adapter Content

## Kmer Content

| Sequence | Count | PValue | Obs/Exp Max | Max Obs/Exp Position |
| --- | --- | --- | --- | --- |
| GTCGCCG | 3850 | 0.0 | 30.631557 | 44-45 |
| CGCCGTA | 4495 | 0.0 | 27.08326 | 46-47 |
| GAGCGGC | 1340 | 0.0 | 26.93654 | 9 |
| TCTCGGG | 1290 | 0.0 | 25.60381 | 36-37 |
| CCGTATC | 4740 | 0.0 | 25.507616 | 48-49 |
| GGCGCCG | 1385 | 0.0 | 25.40739 | 44-45 |
| GGTCGCC | 4225 | 0.0 | 24.535913 | 42-43 |
| GGGCGCC | 1835 | 0.0 | 23.711414 | 42-43 |
| GATCTCG | 6545 | 0.0 | 21.990974 | 34-35 |
| GCCGTAT | 4710 | 0.0 | 21.858868 | 46-47 |
| TCGGGGG | 3445 | 0.0 | 21.181248 | 38-39 |
| GAGGGGC | 1195 | 0.0 | 21.064007 | 9 |
| CGGGAGA | 1200 | 0.0 | 20.946072 | 4 |
| ATCTCGG | 6160 | 0.0 | 20.936335 | 34-35 |
| GTATCAT | 5865 | 0.0 | 20.790289 | 50-51 |
| CGTCGGG | 990 | 0.0 | 20.64606 | 12-13 |
| TCGCCGT | 4585 | 0.0 | 19.03158 | 44-45 |
| CGTATCA | 5340 | 0.0 | 18.90506 | 48-49 |
| GAGCGTC | 12410 | 0.0 | 18.40799 | 9 |
| TCTCGGT | 6425 | 0.0 | 18.15883 | 36-37 |

Produced by FastQC (version 0.11.5)
