# Supplementary material for: Comparison of Mycoplasma pneumoniae Genome Sequences from Strains Isolated from Symptomatic and Asymptomatic Patients
Source: Front Microbiol. 2016 Oct 27;7:1701. doi: 10.3389/fmicb.2016.01701 (PMC5081376; doi:10.3389/fmicb.2016.01701)
Supplement: Supplementary File 1 — Fast QC files. HTML files per strain. Each FastQC report includes: Basic Statistics, Per base sequence, quality, Per sequence quality scores, Per base sequence content, Per sequence GC content, Per base N content, Sequence Length Distribution, Sequence Duplication Levels, Overrepresented sequences, Adapter Content, and Kmer Content. [file DataSheet1.zip › Supplementary files/Supplementary file 1 FastQC/I12-1149-11_interleaved_fastqc.html]

I12-1149-11\_interleaved.fastq FastQC Report 

FastQC Report

Mon 4 Jul 2016  
I12-1149-11\_interleaved.fastq

## Summary

- Basic Statistics
- Per base sequence quality
- Per sequence quality scores
- Per base sequence content
- Per sequence GC content
- Per base N content
- Sequence Length Distribution
- Sequence Duplication Levels
- Overrepresented sequences
- Adapter Content
- Kmer Content

## Basic Statistics

| Measure | Value |
| --- | --- |
| Filename | I12-1149-11\_interleaved.fastq |
| File type | Conventional base calls |
| Encoding | Sanger / Illumina 1.9 |
| Total Sequences | 11295900 |
| Sequences flagged as poor quality | 0 |
| Sequence length | 101 |
| %GC | 39 |

## Per base sequence quality

## Per sequence quality scores

## Per base sequence content

## Per sequence GC content

## Per base N content

## Sequence Length Distribution

## Sequence Duplication Levels

## Overrepresented sequences

| Sequence | Count | Percentage | Possible Source |
| --- | --- | --- | --- |
| GATCGGAAGAGCACACGTCTGAACTCCAGTCACTAGCTTATCTCGTATGC | 109779 | 0.9718481927070884 | TruSeq Adapter, Index 10 (100% over 50bp) |
| GATCGGAAGAGCGTCGTGTAGGGAAAGAGTGTAGATCTCGGTGGTCGCCG | 39786 | 0.3522162908665976 | Illumina Single End PCR Primer 1 (100% over 50bp) |

## Adapter Content

## Kmer Content

| Sequence | Count | PValue | Obs/Exp Max | Max Obs/Exp Position |
| --- | --- | --- | --- | --- |
| GAGCGGC | 3895 | 0.0 | 62.67087 | 9 |
| CGGGAGA | 3235 | 0.0 | 53.942284 | 4 |
| AGAGCGG | 5155 | 0.0 | 50.359776 | 8 |
| GATCGGG | 4545 | 0.0 | 46.981495 | 1 |
| GAGCGTC | 30690 | 0.0 | 43.838917 | 9 |
| GAGGGGC | 2780 | 0.0 | 43.561764 | 9 |
| TCGGGAG | 3615 | 0.0 | 43.029808 | 3 |
| GGAGAGC | 2820 | 0.0 | 42.541218 | 6 |
| GAGAGCG | 2650 | 0.0 | 42.062874 | 7 |
| AGAGCGT | 33440 | 0.0 | 40.973743 | 8 |
| TCTCGGG | 4635 | 0.0 | 40.86095 | 36-37 |
| GGGAGAG | 5155 | 0.0 | 40.840305 | 5 |
| CGGAAGA | 40360 | 0.0 | 40.593143 | 4 |
| TCGGAAG | 39825 | 0.0 | 40.547604 | 3 |
| AAGAGCG | 39140 | 0.0 | 40.306805 | 7 |
| GATCGGA | 42600 | 0.0 | 40.177666 | 1 |
| GTCGCCG | 16510 | 0.0 | 40.00955 | 44-45 |
| GAAGAGC | 40160 | 0.0 | 39.683804 | 6 |
| GAGAGGG | 3455 | 0.0 | 39.264034 | 7 |
| GGCGCCG | 5290 | 0.0 | 39.240726 | 44-45 |

Produced by FastQC (version 0.11.5)
