# Supplementary material for: Comparison of Mycoplasma pneumoniae Genome Sequences from Strains Isolated from Symptomatic and Asymptomatic Patients
Source: Front Microbiol. 2016 Oct 27;7:1701. doi: 10.3389/fmicb.2016.01701 (PMC5081376; doi:10.3389/fmicb.2016.01701)
Supplement: Supplementary File 1 — Fast QC files. HTML files per strain. Each FastQC report includes: Basic Statistics, Per base sequence, quality, Per sequence quality scores, Per base sequence content, Per sequence GC content, Per base N content, Sequence Length Distribution, Sequence Duplication Levels, Overrepresented sequences, Adapter Content, and Kmer Content. [file DataSheet1.zip › Supplementary files/Supplementary file 1 FastQC/I12-1149-12_interleaved_fastqc.html]

I12-1149-12\_interleaved.fastq FastQC Report 

FastQC Report

Mon 4 Jul 2016  
I12-1149-12\_interleaved.fastq

## Summary

- Basic Statistics
- Per base sequence quality
- Per sequence quality scores
- Per base sequence content
- Per sequence GC content
- Per base N content
- Sequence Length Distribution
- Sequence Duplication Levels
- Overrepresented sequences
- Adapter Content
- Kmer Content

## Basic Statistics

| Measure | Value |
| --- | --- |
| Filename | I12-1149-12\_interleaved.fastq |
| File type | Conventional base calls |
| Encoding | Sanger / Illumina 1.9 |
| Total Sequences | 19624370 |
| Sequences flagged as poor quality | 0 |
| Sequence length | 101 |
| %GC | 40 |

## Per base sequence quality

## Per sequence quality scores

## Per base sequence content

## Per sequence GC content

## Per base N content

## Sequence Length Distribution

## Sequence Duplication Levels

## Overrepresented sequences

| Sequence | Count | Percentage | Possible Source |
| --- | --- | --- | --- |
| GATCGGAAGAGCACACGTCTGAACTCCAGTCACGGCTACATCTCGTATGC | 122091 | 0.6221397170966507 | TruSeq Adapter, Index 11 (100% over 50bp) |
| GATCGGAAGAGCGTCGTGTAGGGAAAGAGTGTAGATCTCGGTGGTCGCCG | 47145 | 0.24023701143017587 | Illumina Single End PCR Primer 1 (100% over 50bp) |

## Adapter Content

## Kmer Content

| Sequence | Count | PValue | Obs/Exp Max | Max Obs/Exp Position |
| --- | --- | --- | --- | --- |
| GAGCGGC | 5480 | 0.0 | 50.09183 | 9 |
| CGGGAGA | 4575 | 0.0 | 45.605553 | 4 |
| GAGGGGC | 3420 | 0.0 | 42.770493 | 9 |
| AGAGCGG | 7125 | 0.0 | 41.69983 | 8 |
| TCTCGGG | 5585 | 0.0 | 38.33472 | 36-37 |
| GATCGGG | 6600 | 0.0 | 37.886368 | 1 |
| GTCGCCG | 19370 | 0.0 | 37.51602 | 44-45 |
| GGCGCCG | 6515 | 0.0 | 36.170315 | 44-45 |
| GGGAGAG | 7565 | 0.0 | 36.040676 | 5 |
| GGAGAGC | 4165 | 0.0 | 35.635258 | 6 |
| CGCCGTA | 21155 | 0.0 | 35.23883 | 46-47 |
| GAGAGGG | 4900 | 0.0 | 35.13982 | 7 |
| CCGTATC | 21700 | 0.0 | 35.10304 | 48-49 |
| GAGAGCG | 3925 | 0.0 | 34.925865 | 7 |
| GGGCGCC | 9195 | 0.0 | 34.911762 | 42-43 |
| TCGGGAG | 5425 | 0.0 | 34.61866 | 3 |
| GTATCAT | 22750 | 0.0 | 32.846397 | 50-51 |
| GAGCGTC | 46365 | 0.0 | 32.44999 | 9 |
| GGTCGCC | 20945 | 0.0 | 31.856424 | 42-43 |
| TCGGGGG | 14485 | 0.0 | 31.637758 | 38-39 |

Produced by FastQC (version 0.11.5)
