# Supplementary material for: Comparison of Mycoplasma pneumoniae Genome Sequences from Strains Isolated from Symptomatic and Asymptomatic Patients
Source: Front Microbiol. 2016 Oct 27;7:1701. doi: 10.3389/fmicb.2016.01701 (PMC5081376; doi:10.3389/fmicb.2016.01701)
Supplement: Supplementary File 1 — Fast QC files. HTML files per strain. Each FastQC report includes: Basic Statistics, Per base sequence, quality, Per sequence quality scores, Per base sequence content, Per sequence GC content, Per base N content, Sequence Length Distribution, Sequence Duplication Levels, Overrepresented sequences, Adapter Content, and Kmer Content. [file DataSheet1.zip › Supplementary files/Supplementary file 1 FastQC/I12-1149-13_interleaved_fastqc.html]

I12-1149-13\_interleaved.fastq FastQC Report 

FastQC Report

Mon 4 Jul 2016  
I12-1149-13\_interleaved.fastq

## Summary

- Basic Statistics
- Per base sequence quality
- Per sequence quality scores
- Per base sequence content
- Per sequence GC content
- Per base N content
- Sequence Length Distribution
- Sequence Duplication Levels
- Overrepresented sequences
- Adapter Content
- Kmer Content

## Basic Statistics

| Measure | Value |
| --- | --- |
| Filename | I12-1149-13\_interleaved.fastq |
| File type | Conventional base calls |
| Encoding | Sanger / Illumina 1.9 |
| Total Sequences | 16501684 |
| Sequences flagged as poor quality | 0 |
| Sequence length | 101 |
| %GC | 39 |

## Per base sequence quality

## Per sequence quality scores

## Per base sequence content

## Per sequence GC content

## Per base N content

## Sequence Length Distribution

## Sequence Duplication Levels

## Overrepresented sequences

| Sequence | Count | Percentage | Possible Source |
| --- | --- | --- | --- |
| AGATCGGAAGAGCGTCGTGTAGGGAAAGAGTGTAGATCTCGGTGGTCGCC | 26049 | 0.15785661633079387 | Illumina Single End PCR Primer 1 (100% over 50bp) |
| AGATCGGAAGAGCACACGTCTGAACTCCAGTCACAGTCAACAATCTCGTA | 23790 | 0.14416710439976915 | TruSeq Adapter, Index 13 (97% over 40bp) |
| GATCGGAAGAGCACACGTCTGAACTCCAGTCACAGTCAACAATCTCGTAT | 17730 | 0.10744357969768419 | TruSeq Adapter, Index 13 (97% over 40bp) |

## Adapter Content

## Kmer Content

| Sequence | Count | PValue | Obs/Exp Max | Max Obs/Exp Position |
| --- | --- | --- | --- | --- |
| CCGTATC | 17835 | 0.0 | 24.079842 | 48-49 |
| GTATCAT | 18345 | 0.0 | 23.508982 | 50-51 |
| CGCCGTA | 18700 | 0.0 | 22.59671 | 46-47 |
| GTCGCCG | 18610 | 0.0 | 22.002783 | 44-45 |
| GGCGCCG | 2275 | 0.0 | 20.486391 | 44-45 |
| GCCGTAT | 17950 | 0.0 | 18.235308 | 48-49 |
| TCTCGGG | 2375 | 0.0 | 18.010258 | 36-37 |
| CGTATCA | 18455 | 0.0 | 17.790154 | 50-51 |
| GAGCGGC | 2630 | 0.0 | 17.155064 | 9 |
| TCGGGGG | 4875 | 0.0 | 16.627161 | 38-39 |
| TGGTCGC | 23630 | 0.0 | 16.37234 | 42-43 |
| GGGCGCC | 2935 | 0.0 | 16.041462 | 42-43 |
| TCGCCGT | 19060 | 0.0 | 15.832086 | 46-47 |
| GGTCGCC | 20095 | 0.0 | 15.655345 | 44-45 |
| GAGGGGC | 1730 | 0.0 | 15.647799 | 9 |
| TATCATT | 22425 | 0.0 | 14.928687 | 52-53 |
| GAGAGGG | 2030 | 0.0 | 14.485995 | 7 |
| GGGAGAG | 2985 | 0.0 | 14.296197 | 5 |
| TCGGTGG | 30620 | 0.0 | 14.252986 | 38-39 |
| GGTGGTC | 28175 | 0.0 | 13.941491 | 40-41 |

Produced by FastQC (version 0.11.5)
