# Supplementary material for: Comparison of Mycoplasma pneumoniae Genome Sequences from Strains Isolated from Symptomatic and Asymptomatic Patients
Source: Front Microbiol. 2016 Oct 27;7:1701. doi: 10.3389/fmicb.2016.01701 (PMC5081376; doi:10.3389/fmicb.2016.01701)
Supplement: Supplementary File 1 — Fast QC files. HTML files per strain. Each FastQC report includes: Basic Statistics, Per base sequence, quality, Per sequence quality scores, Per base sequence content, Per sequence GC content, Per base N content, Sequence Length Distribution, Sequence Duplication Levels, Overrepresented sequences, Adapter Content, and Kmer Content. [file DataSheet1.zip › Supplementary files/Supplementary file 1 FastQC/I12-1149-14_interleaved_fastqc.html]

I12-1149-14\_interleaved.fastq FastQC Report 

FastQC Report

Mon 4 Jul 2016  
I12-1149-14\_interleaved.fastq

## Summary

- Basic Statistics
- Per base sequence quality
- Per sequence quality scores
- Per base sequence content
- Per sequence GC content
- Per base N content
- Sequence Length Distribution
- Sequence Duplication Levels
- Overrepresented sequences
- Adapter Content
- Kmer Content

## Basic Statistics

| Measure | Value |
| --- | --- |
| Filename | I12-1149-14\_interleaved.fastq |
| File type | Conventional base calls |
| Encoding | Sanger / Illumina 1.9 |
| Total Sequences | 17740972 |
| Sequences flagged as poor quality | 0 |
| Sequence length | 101 |
| %GC | 39 |

## Per base sequence quality

## Per sequence quality scores

## Per base sequence content

## Per sequence GC content

## Per base N content

## Sequence Length Distribution

## Sequence Duplication Levels

## Overrepresented sequences

| Sequence | Count | Percentage | Possible Source |
| --- | --- | --- | --- |
| GATCGGAAGAGCACACGTCTGAACTCCAGTCACAGTTCCGTATCTCGTAT | 23268 | 0.13115403147020355 | TruSeq Adapter, Index 14 (97% over 44bp) |

## Adapter Content

## Kmer Content

| Sequence | Count | PValue | Obs/Exp Max | Max Obs/Exp Position |
| --- | --- | --- | --- | --- |
| GGCGCCG | 2460 | 0.0 | 22.717524 | 44-45 |
| CCGTATC | 15740 | 0.0 | 21.77643 | 48-49 |
| CGCCGTA | 16060 | 0.0 | 21.175074 | 46-47 |
| TCTCGGG | 2270 | 0.0 | 20.517881 | 36-37 |
| GTCGCCG | 15890 | 0.0 | 20.383627 | 44-45 |
| GTATCAT | 16750 | 0.0 | 20.37784 | 50-51 |
| GAGCGGC | 2630 | 0.0 | 18.95992 | 9 |
| GGGCGCC | 3350 | 0.0 | 18.882635 | 42-43 |
| TCGGGGG | 5745 | 0.0 | 16.799536 | 38-39 |
| GAGAGGG | 1850 | 0.0 | 15.895939 | 7 |
| GAGGGGC | 1680 | 0.0 | 14.981991 | 9 |
| GGGGCCC | 1925 | 0.0 | 14.824455 | 44-45 |
| TGGTCGC | 20550 | 0.0 | 14.696627 | 42-43 |
| CGTCGGG | 2205 | 0.0 | 14.6616335 | 12-13 |
| AGAGCGG | 3960 | 0.0 | 14.141904 | 8 |
| GCCGTAT | 15795 | 0.0 | 13.730179 | 48-49 |
| GGGAGAG | 3225 | 0.0 | 13.526021 | 5 |
| CGGGAGA | 2490 | 0.0 | 13.520317 | 4 |
| CTCGGGG | 4235 | 0.0 | 13.466667 | 36-37 |
| CGTATCA | 16505 | 0.0 | 13.250357 | 50-51 |

Produced by FastQC (version 0.11.5)
