# Supplementary material for: Comparison of Mycoplasma pneumoniae Genome Sequences from Strains Isolated from Symptomatic and Asymptomatic Patients
Source: Front Microbiol. 2016 Oct 27;7:1701. doi: 10.3389/fmicb.2016.01701 (PMC5081376; doi:10.3389/fmicb.2016.01701)
Supplement: Supplementary File 1 — Fast QC files. HTML files per strain. Each FastQC report includes: Basic Statistics, Per base sequence, quality, Per sequence quality scores, Per base sequence content, Per sequence GC content, Per base N content, Sequence Length Distribution, Sequence Duplication Levels, Overrepresented sequences, Adapter Content, and Kmer Content. [file DataSheet1.zip › Supplementary files/Supplementary file 1 FastQC/I12-1149-15_interleaved_fastqc.html]

I12-1149-15\_interleaved.fastq FastQC Report 

FastQC Report

Mon 4 Jul 2016  
I12-1149-15\_interleaved.fastq

## Summary

- Basic Statistics
- Per base sequence quality
- Per sequence quality scores
- Per base sequence content
- Per sequence GC content
- Per base N content
- Sequence Length Distribution
- Sequence Duplication Levels
- Overrepresented sequences
- Adapter Content
- Kmer Content

## Basic Statistics

| Measure | Value |
| --- | --- |
| Filename | I12-1149-15\_interleaved.fastq |
| File type | Conventional base calls |
| Encoding | Sanger / Illumina 1.9 |
| Total Sequences | 13941048 |
| Sequences flagged as poor quality | 0 |
| Sequence length | 101 |
| %GC | 40 |

## Per base sequence quality

## Per sequence quality scores

## Per base sequence content

## Per sequence GC content

## Per base N content

## Sequence Length Distribution

## Sequence Duplication Levels

## Overrepresented sequences

| Sequence | Count | Percentage | Possible Source |
| --- | --- | --- | --- |
| GATCGGAAGAGCACACGTCTGAACTCCAGTCACATGTCAGAATCTCGTAT | 82211 | 0.5897045903579128 | TruSeq Adapter, Index 15 (97% over 40bp) |
| AGATCGGAAGAGCGTCGTGTAGGGAAAGAGTGTAGATCTCGGTGGTCGCC | 43619 | 0.3128817862186544 | Illumina Single End PCR Primer 1 (100% over 50bp) |
| AGATCGGAAGAGCACACGTCTGAACTCCAGTCACATGTCAGAATCTCGTA | 38360 | 0.2751586537826999 | TruSeq Adapter, Index 15 (97% over 40bp) |
| GATCGGAAGAGCGTCGTGTAGGGAAAGAGTGTAGATCTCGGTGGTCGCCG | 32364 | 0.2321489747399191 | Illumina Single End PCR Primer 1 (100% over 50bp) |

## Adapter Content

## Kmer Content

| Sequence | Count | PValue | Obs/Exp Max | Max Obs/Exp Position |
| --- | --- | --- | --- | --- |
| GAGCGGC | 4180 | 0.0 | 46.92276 | 9 |
| CGGGAGA | 3215 | 0.0 | 42.32602 | 4 |
| GAGGGGC | 2390 | 0.0 | 39.939987 | 9 |
| AGAGCGG | 5455 | 0.0 | 38.194042 | 8 |
| GATCGGG | 5010 | 0.0 | 37.216377 | 1 |
| GGCGCCG | 5760 | 0.0 | 35.547333 | 44-45 |
| GGGAGAG | 4955 | 0.0 | 35.020485 | 5 |
| TCTCGGG | 4870 | 0.0 | 34.69557 | 36-37 |
| GAGAGGG | 3295 | 0.0 | 33.539623 | 7 |
| TCGGGAG | 3725 | 0.0 | 33.098484 | 3 |
| GAGAGCG | 2715 | 0.0 | 32.319126 | 7 |
| GGAGAGC | 2950 | 0.0 | 32.304184 | 6 |
| TCGGGGG | 10835 | 0.0 | 31.550278 | 38-39 |
| GGGCGCC | 7895 | 0.0 | 30.48268 | 42-43 |
| GGAGAGG | 3795 | 0.0 | 29.608833 | 6 |
| CCGTATC | 35660 | 0.0 | 29.555883 | 48-49 |
| GTATCAT | 35645 | 0.0 | 29.345934 | 50-51 |
| CGTCGGG | 3790 | 0.0 | 28.851913 | 12-13 |
| CGCCGTA | 36410 | 0.0 | 28.64704 | 46-47 |
| CGCCGGA | 4660 | 0.0 | 28.169876 | 46-47 |

Produced by FastQC (version 0.11.5)
