# Supplementary material for: Comparison of Mycoplasma pneumoniae Genome Sequences from Strains Isolated from Symptomatic and Asymptomatic Patients
Source: Front Microbiol. 2016 Oct 27;7:1701. doi: 10.3389/fmicb.2016.01701 (PMC5081376; doi:10.3389/fmicb.2016.01701)
Supplement: Supplementary File 1 — Fast QC files. HTML files per strain. Each FastQC report includes: Basic Statistics, Per base sequence, quality, Per sequence quality scores, Per base sequence content, Per sequence GC content, Per base N content, Sequence Length Distribution, Sequence Duplication Levels, Overrepresented sequences, Adapter Content, and Kmer Content. [file DataSheet1.zip › Supplementary files/Supplementary file 1 FastQC/I12-1149-16_interleaved_fastqc.html]

I12-1149-16\_interleaved.fastq FastQC Report 

FastQC Report

Mon 4 Jul 2016  
I12-1149-16\_interleaved.fastq

## Summary

- Basic Statistics
- Per base sequence quality
- Per sequence quality scores
- Per base sequence content
- Per sequence GC content
- Per base N content
- Sequence Length Distribution
- Sequence Duplication Levels
- Overrepresented sequences
- Adapter Content
- Kmer Content

## Basic Statistics

| Measure | Value |
| --- | --- |
| Filename | I12-1149-16\_interleaved.fastq |
| File type | Conventional base calls |
| Encoding | Sanger / Illumina 1.9 |
| Total Sequences | 16570952 |
| Sequences flagged as poor quality | 0 |
| Sequence length | 101 |
| %GC | 40 |

## Per base sequence quality

## Per sequence quality scores

## Per base sequence content

## Per sequence GC content

## Per base N content

## Sequence Length Distribution

## Sequence Duplication Levels

## Overrepresented sequences

| Sequence | Count | Percentage | Possible Source |
| --- | --- | --- | --- |
| GATCGGAAGAGCACACGTCTGAACTCCAGTCACCCGTCCCGATCTCGTAT | 39985 | 0.24129573243589142 | TruSeq Adapter, Index 16 (97% over 40bp) |
| GATCGGAAGAGCGTCGTGTAGGGAAAGAGTGTAGATCTCGGTGGTCGCCG | 16765 | 0.10117101298706314 | Illumina Single End PCR Primer 1 (100% over 50bp) |

## Adapter Content

## Kmer Content

| Sequence | Count | PValue | Obs/Exp Max | Max Obs/Exp Position |
| --- | --- | --- | --- | --- |
| GTCGCCG | 10610 | 0.0 | 37.272793 | 44-45 |
| CGCCGTA | 12325 | 0.0 | 33.534443 | 46-47 |
| GAGCGGC | 3290 | 0.0 | 33.19978 | 9 |
| CCGTATC | 12855 | 0.0 | 32.409863 | 48-49 |
| TCTCGGG | 2500 | 0.0 | 32.03366 | 36-37 |
| GTATCAT | 14205 | 0.0 | 29.255415 | 50-51 |
| GGCGCCG | 3150 | 0.0 | 28.76269 | 44-45 |
| GATCTCG | 16805 | 0.0 | 28.465382 | 34-35 |
| AGAGCGG | 4355 | 0.0 | 26.807333 | 8 |
| GGGCGCC | 4410 | 0.0 | 26.206528 | 42-43 |
| TCTCGGT | 15450 | 0.0 | 26.117157 | 36-37 |
| TGGTCGC | 14315 | 0.0 | 25.416273 | 42-43 |
| TAGATCT | 18855 | 0.0 | 23.786858 | 32-33 |
| TCGGTGG | 18095 | 0.0 | 23.620035 | 38-39 |
| CGGGAGA | 2835 | 0.0 | 23.248556 | 4 |
| TCGGGGG | 7265 | 0.0 | 22.249628 | 38-39 |
| GAGAGGG | 2550 | 0.0 | 21.947159 | 7 |
| GGTCGCC | 11690 | 0.0 | 21.705095 | 42-43 |
| GAGGGGC | 2175 | 0.0 | 21.616194 | 9 |
| AGAGTGT | 18165 | 0.0 | 21.189707 | 26-27 |

Produced by FastQC (version 0.11.5)
