# Supplementary material for: Comparison of Mycoplasma pneumoniae Genome Sequences from Strains Isolated from Symptomatic and Asymptomatic Patients
Source: Front Microbiol. 2016 Oct 27;7:1701. doi: 10.3389/fmicb.2016.01701 (PMC5081376; doi:10.3389/fmicb.2016.01701)
Supplement: Supplementary File 1 — Fast QC files. HTML files per strain. Each FastQC report includes: Basic Statistics, Per base sequence, quality, Per sequence quality scores, Per base sequence content, Per sequence GC content, Per base N content, Sequence Length Distribution, Sequence Duplication Levels, Overrepresented sequences, Adapter Content, and Kmer Content. [file DataSheet1.zip › Supplementary files/Supplementary file 1 FastQC/I12-1149-17_interleaved_fastqc.html]

I12-1149-17\_interleaved.fastq FastQC Report 

FastQC Report

Mon 4 Jul 2016  
I12-1149-17\_interleaved.fastq

## Summary

- Basic Statistics
- Per base sequence quality
- Per sequence quality scores
- Per base sequence content
- Per sequence GC content
- Per base N content
- Sequence Length Distribution
- Sequence Duplication Levels
- Overrepresented sequences
- Adapter Content
- Kmer Content

## Basic Statistics

| Measure | Value |
| --- | --- |
| Filename | I12-1149-17\_interleaved.fastq |
| File type | Conventional base calls |
| Encoding | Sanger / Illumina 1.9 |
| Total Sequences | 16202678 |
| Sequences flagged as poor quality | 0 |
| Sequence length | 101 |
| %GC | 39 |

## Per base sequence quality

## Per sequence quality scores

## Per base sequence content

## Per sequence GC content

## Per base N content

## Sequence Length Distribution

## Sequence Duplication Levels

## Overrepresented sequences

No overrepresented sequences

## Adapter Content

## Kmer Content

| Sequence | Count | PValue | Obs/Exp Max | Max Obs/Exp Position |
| --- | --- | --- | --- | --- |
| GTCGCCG | 2200 | 0.0 | 25.941425 | 44-45 |
| CGCCGTA | 3785 | 0.0 | 15.895272 | 46-47 |
| TCTCGGG | 1060 | 0.0 | 13.8990345 | 36-37 |
| CCGTATC | 4410 | 0.0 | 13.896238 | 48-49 |
| GATCTCG | 5105 | 0.0 | 13.8640785 | 34-35 |
| GGTCGCC | 2935 | 0.0 | 13.854469 | 42-43 |
| GGCGCCG | 1250 | 0.0 | 12.555651 | 44-45 |
| ATCTCGG | 4075 | 0.0 | 12.181174 | 34-35 |
| GCCGTAT | 3915 | 0.0 | 10.811889 | 46-47 |
| GTATCAT | 5800 | 0.0 | 10.388466 | 50-51 |
| TCTCGGT | 5635 | 0.0 | 10.120831 | 36-37 |
| GGGCGCC | 1990 | 0.0 | 9.79857 | 42-43 |
| TGGTCGC | 5910 | 0.0 | 9.053093 | 42-43 |
| GGGGCCC | 1135 | 0.0 | 9.009027 | 44-45 |
| TAGATCT | 7860 | 0.0 | 8.962476 | 32-33 |
| AGAGTGT | 6910 | 0.0 | 8.757746 | 26-27 |
| GAGCGTC | 10230 | 0.0 | 8.634671 | 9 |
| TCGCCGT | 4390 | 0.0 | 8.287665 | 44-45 |
| AGTGTAG | 7290 | 0.0 | 8.200928 | 28-29 |
| TCGGTGG | 7805 | 0.0 | 8.040027 | 38-39 |

Produced by FastQC (version 0.11.5)
