# Supplementary material for: Comparison of Mycoplasma pneumoniae Genome Sequences from Strains Isolated from Symptomatic and Asymptomatic Patients
Source: Front Microbiol. 2016 Oct 27;7:1701. doi: 10.3389/fmicb.2016.01701 (PMC5081376; doi:10.3389/fmicb.2016.01701)
Supplement: Supplementary File 1 — Fast QC files. HTML files per strain. Each FastQC report includes: Basic Statistics, Per base sequence, quality, Per sequence quality scores, Per base sequence content, Per sequence GC content, Per base N content, Sequence Length Distribution, Sequence Duplication Levels, Overrepresented sequences, Adapter Content, and Kmer Content. [file DataSheet1.zip › Supplementary files/Supplementary file 1 FastQC/I12-1149-18_interleaved_fastqc.html]

I12-1149-18\_interleaved.fastq FastQC Report 

FastQC Report

Mon 4 Jul 2016  
I12-1149-18\_interleaved.fastq

## Summary

- Basic Statistics
- Per base sequence quality
- Per sequence quality scores
- Per base sequence content
- Per sequence GC content
- Per base N content
- Sequence Length Distribution
- Sequence Duplication Levels
- Overrepresented sequences
- Adapter Content
- Kmer Content

## Basic Statistics

| Measure | Value |
| --- | --- |
| Filename | I12-1149-18\_interleaved.fastq |
| File type | Conventional base calls |
| Encoding | Sanger / Illumina 1.9 |
| Total Sequences | 18897466 |
| Sequences flagged as poor quality | 0 |
| Sequence length | 101 |
| %GC | 40 |

## Per base sequence quality

## Per sequence quality scores

## Per base sequence content

## Per sequence GC content

## Per base N content

## Sequence Length Distribution

## Sequence Duplication Levels

## Overrepresented sequences

| Sequence | Count | Percentage | Possible Source |
| --- | --- | --- | --- |
| GATCGGAAGAGCACACGTCTGAACTCCAGTCACGTGAAACGATCTCGTAT | 22864 | 0.12098976656446955 | TruSeq Adapter, Index 19 (97% over 40bp) |

## Adapter Content

## Kmer Content

| Sequence | Count | PValue | Obs/Exp Max | Max Obs/Exp Position |
| --- | --- | --- | --- | --- |
| GTCGCCG | 5535 | 0.0 | 30.974363 | 44-45 |
| TCTCGGG | 2065 | 0.0 | 25.087759 | 36-37 |
| CGCCGTA | 7315 | 0.0 | 24.218287 | 46-47 |
| GGTCGCC | 6660 | 0.0 | 23.88531 | 42-43 |
| GGCGCCG | 2355 | 0.0 | 23.62706 | 44-45 |
| CCGTATC | 7785 | 0.0 | 23.616648 | 48-49 |
| ATCTCGG | 8970 | 0.0 | 22.402561 | 34-35 |
| GCCGTAT | 7315 | 0.0 | 21.845219 | 46-47 |
| GAGCGGC | 2830 | 0.0 | 21.145174 | 9 |
| GATCTCG | 10435 | 0.0 | 21.123959 | 34-35 |
| GGGCGCC | 3575 | 0.0 | 20.685371 | 42-43 |
| GTATCAT | 9320 | 0.0 | 19.445156 | 50-51 |
| TCGCCGT | 7500 | 0.0 | 18.07167 | 44-45 |
| CGTATCA | 8875 | 0.0 | 17.879032 | 48-49 |
| TCTCGGT | 10210 | 0.0 | 17.340273 | 36-37 |
| TCGGGGG | 5910 | 0.0 | 16.851828 | 38-39 |
| AGATCTC | 12150 | 0.0 | 16.671217 | 32-33 |
| AGAGCGG | 4085 | 0.0 | 16.498253 | 8 |
| CGGGAGA | 2550 | 0.0 | 16.362753 | 4 |
| CGTCGGG | 2070 | 0.0 | 16.306032 | 12-13 |

Produced by FastQC (version 0.11.5)
