# Supplementary material for: Comparison of Mycoplasma pneumoniae Genome Sequences from Strains Isolated from Symptomatic and Asymptomatic Patients
Source: Front Microbiol. 2016 Oct 27;7:1701. doi: 10.3389/fmicb.2016.01701 (PMC5081376; doi:10.3389/fmicb.2016.01701)
Supplement: Supplementary File 1 — Fast QC files. HTML files per strain. Each FastQC report includes: Basic Statistics, Per base sequence, quality, Per sequence quality scores, Per base sequence content, Per sequence GC content, Per base N content, Sequence Length Distribution, Sequence Duplication Levels, Overrepresented sequences, Adapter Content, and Kmer Content. [file DataSheet1.zip › Supplementary files/Supplementary file 1 FastQC/I12-1149-19_interleaved_fastqc.html]

I12-1149-19\_interleaved.fastq FastQC Report 

FastQC Report

Mon 4 Jul 2016  
I12-1149-19\_interleaved.fastq

## Summary

- Basic Statistics
- Per base sequence quality
- Per sequence quality scores
- Per base sequence content
- Per sequence GC content
- Per base N content
- Sequence Length Distribution
- Sequence Duplication Levels
- Overrepresented sequences
- Adapter Content
- Kmer Content

## Basic Statistics

| Measure | Value |
| --- | --- |
| Filename | I12-1149-19\_interleaved.fastq |
| File type | Conventional base calls |
| Encoding | Sanger / Illumina 1.9 |
| Total Sequences | 14742908 |
| Sequences flagged as poor quality | 0 |
| Sequence length | 101 |
| %GC | 40 |

## Per base sequence quality

## Per sequence quality scores

## Per base sequence content

## Per sequence GC content

## Per base N content

## Sequence Length Distribution

## Sequence Duplication Levels

## Overrepresented sequences

| Sequence | Count | Percentage | Possible Source |
| --- | --- | --- | --- |
| GATCGGAAGAGCACACGTCTGAACTCCAGTCACCGTACGTAATCTCGTAT | 91399 | 0.6199523187691329 | TruSeq Adapter, Index 22 (97% over 40bp) |
| GATCGGAAGAGCGTCGTGTAGGGAAAGAGTGTAGATCTCGGTGGTCGCCG | 36984 | 0.25085959974789235 | Illumina Single End PCR Primer 1 (100% over 50bp) |

## Adapter Content

## Kmer Content

| Sequence | Count | PValue | Obs/Exp Max | Max Obs/Exp Position |
| --- | --- | --- | --- | --- |
| GAGCGGC | 4035 | 0.0 | 53.897984 | 9 |
| AGAGCGG | 5255 | 0.0 | 44.157707 | 8 |
| CGGGAGA | 3400 | 0.0 | 43.93287 | 4 |
| GAGGGGC | 2835 | 0.0 | 40.1984 | 9 |
| GTCGCCG | 17040 | 0.0 | 40.191723 | 44-45 |
| TCTCGGG | 4535 | 0.0 | 38.249542 | 36-37 |
| GAGAGGG | 3585 | 0.0 | 38.233944 | 7 |
| GATCGGG | 5105 | 0.0 | 38.105507 | 1 |
| CCGTATC | 19015 | 0.0 | 37.83508 | 48-49 |
| CGCCGTA | 18730 | 0.0 | 37.745735 | 46-47 |
| GGCGCCG | 5180 | 0.0 | 37.50646 | 44-45 |
| GGGAGAG | 5310 | 0.0 | 37.326786 | 5 |
| GGGCGCC | 7295 | 0.0 | 35.987553 | 42-43 |
| GGAGAGC | 2795 | 0.0 | 35.626633 | 6 |
| GTATCAT | 19925 | 0.0 | 35.392654 | 50-51 |
| GATCTCG | 24180 | 0.0 | 35.014603 | 34-35 |
| TCGGGAG | 3945 | 0.0 | 34.981804 | 3 |
| GAGCGTC | 35015 | 0.0 | 34.28258 | 9 |
| GAGAGCG | 2915 | 0.0 | 32.70378 | 7 |
| TCTCGGT | 21065 | 0.0 | 32.690205 | 36-37 |

Produced by FastQC (version 0.11.5)
