# Supplementary material for: Comparison of Mycoplasma pneumoniae Genome Sequences from Strains Isolated from Symptomatic and Asymptomatic Patients
Source: Front Microbiol. 2016 Oct 27;7:1701. doi: 10.3389/fmicb.2016.01701 (PMC5081376; doi:10.3389/fmicb.2016.01701)
Supplement: Supplementary File 1 — Fast QC files. HTML files per strain. Each FastQC report includes: Basic Statistics, Per base sequence, quality, Per sequence quality scores, Per base sequence content, Per sequence GC content, Per base N content, Sequence Length Distribution, Sequence Duplication Levels, Overrepresented sequences, Adapter Content, and Kmer Content. [file DataSheet1.zip › Supplementary files/Supplementary file 1 FastQC/I12-1149-20_interleaved_fastqc.html]

I12-1149-20\_interleaved.fastq FastQC Report 

FastQC Report

Mon 4 Jul 2016  
I12-1149-20\_interleaved.fastq

## Summary

- Basic Statistics
- Per base sequence quality
- Per sequence quality scores
- Per base sequence content
- Per sequence GC content
- Per base N content
- Sequence Length Distribution
- Sequence Duplication Levels
- Overrepresented sequences
- Adapter Content
- Kmer Content

## Basic Statistics

| Measure | Value |
| --- | --- |
| Filename | I12-1149-20\_interleaved.fastq |
| File type | Conventional base calls |
| Encoding | Sanger / Illumina 1.9 |
| Total Sequences | 16694932 |
| Sequences flagged as poor quality | 0 |
| Sequence length | 101 |
| %GC | 39 |

## Per base sequence quality

## Per sequence quality scores

## Per base sequence content

## Per sequence GC content

## Per base N content

## Sequence Length Distribution

## Sequence Duplication Levels

## Overrepresented sequences

No overrepresented sequences

## Adapter Content

## Kmer Content

| Sequence | Count | PValue | Obs/Exp Max | Max Obs/Exp Position |
| --- | --- | --- | --- | --- |
| GTCGCCG | 2475 | 0.0 | 25.17197 | 44-45 |
| CGCCGTA | 3845 | 0.0 | 17.007221 | 46-47 |
| CCGTATC | 4575 | 0.0 | 14.589101 | 48-49 |
| TCTCGGG | 1005 | 0.0 | 14.423153 | 36-37 |
| GGCGCCG | 1185 | 0.0 | 14.247253 | 44-45 |
| GATCTCG | 5850 | 0.0 | 13.43808 | 34-35 |
| GGTCGCC | 3125 | 0.0 | 13.01166 | 42-43 |
| GTATCAT | 6030 | 0.0 | 11.762122 | 50-51 |
| ATCTCGG | 4550 | 0.0 | 11.274764 | 34-35 |
| GGGCGCC | 1945 | 0.0 | 10.880704 | 42-43 |
| GCCGTAT | 4030 | 0.0 | 10.502967 | 46-47 |
| TCTCGGT | 6615 | 0.0 | 10.453447 | 36-37 |
| TGGTCGC | 6625 | 0.0 | 9.296094 | 42-43 |
| TAGATCT | 8470 | 0.0 | 8.933109 | 32-33 |
| CGTCGGG | 1295 | 0.0 | 8.626942 | 12-13 |
| AGAGTGT | 7345 | 0.0 | 8.626762 | 26-27 |
| TCGGTGG | 8675 | 0.0 | 8.603825 | 38-39 |
| GCGTCGG | 1205 | 0.0 | 8.477231 | 10-11 |
| GAGCGTC | 11170 | 0.0 | 8.332728 | 9 |
| TCGCCGT | 4510 | 0.0 | 8.277788 | 44-45 |

Produced by FastQC (version 0.11.5)
