# Supplementary figures and images for: Comparison of Mycoplasma pneumoniae Genome Sequences from Strains Isolated from Symptomatic and Asymptomatic Patients
Source: Front Microbiol. 2016 Oct 27;7:1701. doi: 10.3389/fmicb.2016.01701 (PMC5081376; doi:10.3389/fmicb.2016.01701)

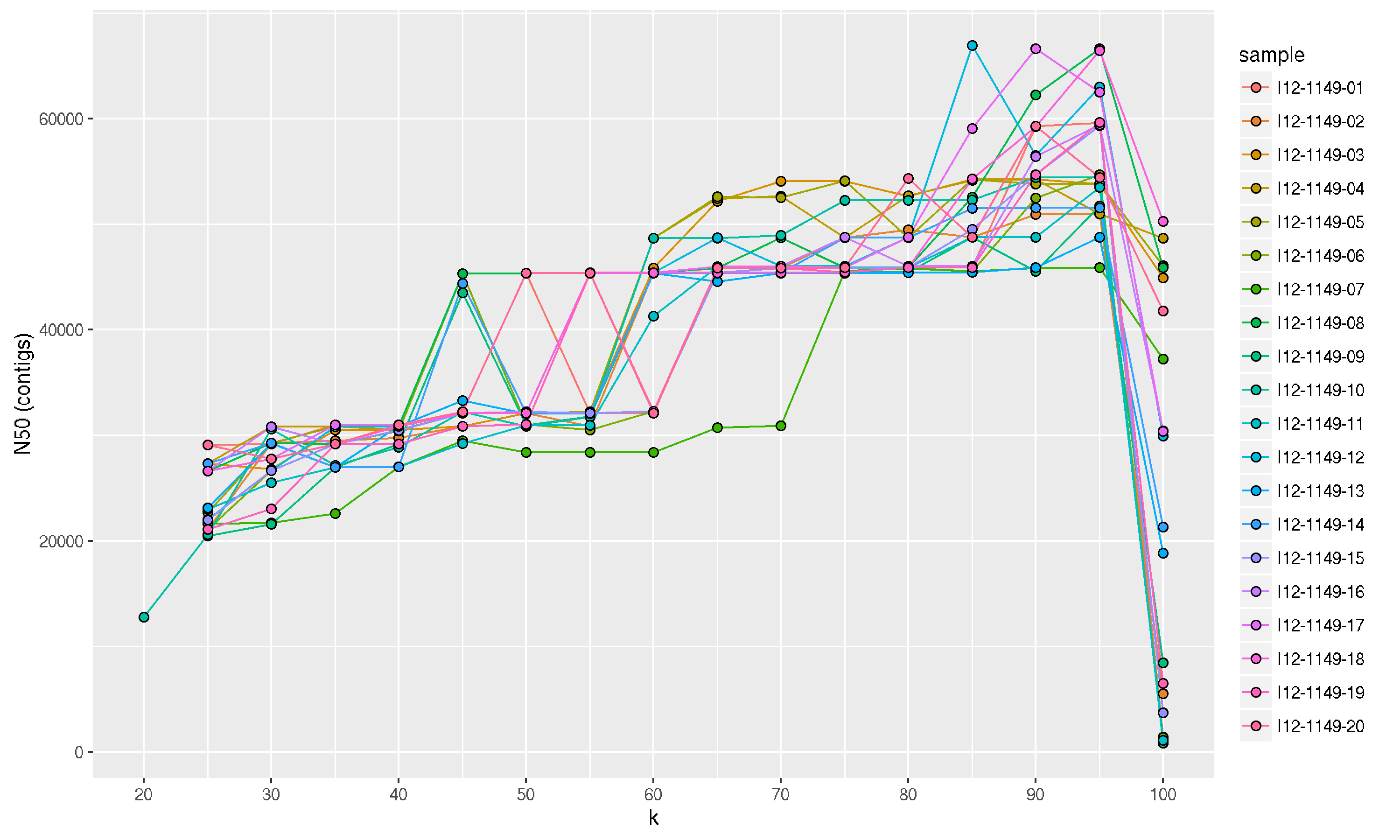

Supplement: Supplementary Figure 1 — The results of the optimization on the assembly statistics. [file Image1.PNG]
